# EEG biomarkers of the sense of embodiment: methodological gaps and evidence-based recommendations from a systematic review

**DOI:** 10.3389/fnsys.2026.1756407

**Published:** 2026-03-20

**Authors:** Daniela Esteves, Athanasios Vourvopoulos

**Affiliations:** Institute for Systems and Robotics (ISR-Lisboa), Bioengineering Department, Instituto Superior Técnico, Lisbon, Portugal

**Keywords:** brain-computer interfaces, electroencephalography, neural biomarkers, sense of embodiment, virtual reality

## Abstract

**Introduction:**

The sense of embodiment (SoE), describing the experience of owning, controlling, and being located within a body, underpins virtual reality (VR) interaction, brain-computer interfaces (BCIs), and multisensory body-illusion research. Although SoE is typically assessed through subjective questionnaires, their variability and limited validity have motivated the search for objective neural markers. Electroencephalography (EEG) has become the most widely used technique given its portability and high temporal resolution; however, the existence of a consistent EEG correlate of embodiment remains unclear.

**Methods:**

This systematic review summarizes 35 EEG studies (2010–June 2025) identified through structured database searches, examining SoE across immersive and non-immersive VR, augmented reality, and non-VR paradigms. We analyze EEG features including spectral power, event-related desynchronization/synchronization (ERD/ERS), connectivity, and temporal dynamics, and examine methodological variability in illusion induction and SoE assessment.

**Results:**

Across studies, the reduction of the alpha-band over central-parietal regions emerges as the most recurrent correlate of embodiment. Beta-band decreases and gamma-band increases appear in several studies but lack consistent replication, while findings in Delta and Theta bands remain sparse and contradictory. Considerable heterogeneity is found in VR paradigms, EEG setups, preprocessing, and psychometric tools, contributing to inconsistent results and limiting cross-study comparability.

**Discussion:**

Critically, no EEG feature demonstrates sufficient reproducibility to qualify as a universal biomarker of SoE, and no standardized protocol for EEG-based embodiment assessment currently exists. Overall, this review highlights both the promise and current limitations of EEG-based approaches to measuring embodiment. We conclude by identifying methodological gaps and outlining recommendations to support the development of reliable EEG markers for future applications in VR rehabilitation, MI-BCIs, cognitive neuroscience, and clinical interventions.

## 1 Introduction

Sense of Embodiment (SoE) refers to the subjective perception that a non-biological body or body part, such as a virtual avatar or prosthetic limb, feels like one's own body. This arises from the alignment of sensations of being within, owning, and controlling the artificial body part (Kilteni et al., 2012a). The concept was first introduced by Botvinick and Cohen (1998) through the well-known Rubber Hand Illusion (RHI), which demonstrated that tactile sensations could be transferred to an artificial limb. In this paradigm, participants observed a rubber hand being stroked while their real, hidden hand was touched synchronously. This multisensory congruence led participants to perceive the rubber hand as their own, making the RHI a foundational experiment for exploring body ownership and the brain's construction of self-perception.

Following the original RHI, numerous studies have replicated and extended this phenomenon using different sensory feedback modalities. Among the most powerful tools for inducing SoE illusion is Virtual Reality (VR), since it enables the replacement of the user's real body with a virtual avatar, allowing the controlled manipulation of SoE (Guy et al., 2023). VR can simulate realistic or fantastical computer-generated scenarios in which users can interact with and experience a virtual body, making it an ideal platform for controlled embodiment illusions. This effect is further enhanced by VR's capacity to evoke a strong sense of immersion, typically achieved through head-mounted displays (HMDs). Immersive VR, characterized by 3D environments, is distinguished from non-immersive VR by its ability to create a sense of physical presence, which is the feeling of being within the virtual world, independent of body perception (Dincelli and Yayla, 2022; Kilteni et al., 2012a; Guy et al., 2023). These controlled environments make VR an ideal setting for inducing embodiment illusions through multisensory feedback, often involving visuoproprioceptive cues, motion tracking, and haptic interaction. Visual feedback, in particular, plays a crucial role, especially when the virtual body responds synchronously to the user's voluntary movements (Choi et al., 2020b; Škola and Liarokapis, 2023). This coupling of sensory input and motor output enhances SoE, creating a rich and immersive experience (Ferreira, 2006; Vourvopoulos et al., 2022, 2016). Early studies extended the RHI into VR (Lenggenhager et al., 2007), later advancing to full-body ownership illusions with applications in rehabilitation, gaming, and neuroscience (Slater, 2009; Petkova and Ehrsson, 2008; Vagaja et al., 2024; Guy et al., 2023).

With the increasing accessibility of immersive VR technologies, virtual embodiment has been applied across multiple healthcare domains. Pérez-Marcos et al. (2009) were the first to demonstrate that SoE over a virtual hand could be induced using motor imagery (MI) via a brain-computer interface (BCI). MI-BCI systems work by recording brain activity generated during MI tasks, that is, when individuals mentally rehearse movements without physically executing them (Chen et al., 2023; Pichiorri et al., 2015; Gu et al., 2021). This neural information is then translated into commands for external devices, such as robotic arms or, in this case, a virtual avatar, allowing them to act on the user's behalf (Wolpaw et al., 2020; Chen et al., 2023; Daly and Huggins, 2015). In the study by Pérez-Marcos et al. (2009), neurofeedback was provided by rendering a virtual hand that performed the imagined movement in synchrony with the user's intention, effectively reinforcing the SoE over the virtual limb. This integration of MI and VR opens promising pathways for immersive, user-centered rehabilitation therapies. Based on this, in neurorehabilitation, integrating embodiment into MI-BCI training has been shown to enhance patients'ability to modulate brain activity and improve recovery outcomes (Vagaja et al., 2024; Batista et al., 2024; Juliano et al., 2020; Choi et al., 2020a), leading to a growing interest in SoE. Virtual embodiment and embodiment have been used in psychiatric and psychological treatments, including exposure therapy for phobias (Tardif et al., 2019), therapy for eating disorders (Cesa et al., 2013), treatment of post-traumatic stress disorder (PTSD) (Reger and Gahm, 2008), and pain management for burn patients (Hoffman et al., 2000). Embodiment-based feedback has also been employed in psychological self-counseling programs (Osimo et al., 2015). Moreover, SoE provides insights into neurological disorders. For instance, in anarchic hand syndrome, patients perceive their limb as acting independently of their will, reflecting a breakdown in SoE (Kilteni et al., 2012a).

Given its broad potential in multiple healthcare areas, embodiment illusion has grown considerably and, as a result, so has its conceptual framework. The most recent and widely adopted model defines SoE as comprising three interrelated components: Sense of Ownership (SoO), Sense of Agency (SoA), and Sense of Self-location (SoSL). When these three components are aligned, a strong SoE toward a virtual body or object is typically established (Kilteni et al., 2012a; Vagaja et al., 2024; Guy et al., 2023).

SoO refers to self-attribution of the body, of the belief that the experienced sensations are being applied to the individual's body. In the VR community, the distinction between ownership of the whole body or specific body parts is less pronounced since the focus often lies on embodying full-body avatars (Kilteni et al., 2012a; Segil et al., 2022; Guy et al., 2023). SoO was the primary effect observed during the RHI and it became the first component of the SoE to be extensively studied (Guy et al., 2023). It arises from bottom-up sensory inputs (e.g., visual, tactile, and proprioceptive) and top-down cognitive expectations (e.g., internal body maps). Essentially, it refers to how the brain processes and interprets sensory input based on expectations. SoO only occurs when both these influences are aligned, creating a coherent perception of the body. However, how these processes interact is still unclear (Kilteni et al., 2012a; Guy et al., 2023). Additionally, research suggests that stronger SoO is induced when external objects resemble the real body, requiring anatomical plausibility and spatial alignment. VR enhances this by enabling customizable avatars that closely match users' real bodies (Guy et al., 2023; Segil et al., 2022).

SoA is the experience of controlling one's movements and their outcomes. It includes the feeling of agency, an implicit, low-level, non-reflective sense tied to action initiation, and the judgment of agency, which is a higher-level, reflective reasoning of the action based on sensory feedback. Thus, this sense arises from sensorimotor integration and cognitive processes. SoA is triggered when predicted and actual sensory feedback align, with temporal and spatial congruence being critical (Kilteni et al., 2012a; Segil et al., 2022; Guy et al., 2023). In VR, immersive tracking and low-latency feedback enhance SoA, ensuring seamless control of virtual avatars (Guy et al., 2023).

Finally, SoSL refers to the subjective experience of perceiving oneself as situated within one's body, with a clear awareness of spatial position and the physical boundaries separating the body from the surrounding environment. It is shaped by visuospatial perspective (viewing the world from within the body or from an external vantage point), vestibular signals (providing information about motion, rotation, balance, and orientation), and tactile input. Tactile stimulation operates across three spatial zones: personal space (the skin and the body's boundaries), peripersonal space (within arm's reach), and extrapersonal space (beyond arm's reach). Interestingly, studies have shown that individuals can experience self-location in two different places simultaneously, adding further complexity to the understanding of SoSL (Kilteni et al., 2012a; Guy et al., 2023).

Research has shown that the three components of SoE are distinct and can be experienced independently, yet they remain deeply interconnected. VR provides unique opportunities to examine SoO, SoA, and SoSL in isolation; however, these components are not easily disentangled at the neurophysiological level, which complicates the understanding of SoE. Moreover, little is known about how each subcomponent contributes to the overall experience or how they interact with one another. Importantly, the relative significance of each component appears to vary across experimental contexts (Kilteni et al., 2012a; Guy et al., 2023; Segil et al., 2022).

The induction of SoE relies on sensory cues that align with SoO, SoA, and SoSL. In VR, researchers typically use three primary types of sensory triggers: visuomotor (synchronization between visual feedback and motor execution), visuotactile (alignment between the tactile sensations perceived and the visual feedback received from the virtual body), and visuoproprioceptive (visuospatial perspective) (Vagaja et al., 2024; Guy et al., 2023). Additional influences include task goals, emotions, personality traits, and social or cultural factors. Users often adjust their behavior and elements of a virtual avatar (control, appearance, and perspective) based on their task goals and personal inclinations. This effect, called the Proteus effect, leads to users modifying their actions and cognitive performance based on the characteristics of their avatars (Yee and Bailenson, 2007; Peck et al., 2013; Banakou et al., 2016, 2018). However, it's essential to note that these embodiment effects are not universal. While some individuals may experience profound behavioral changes, such as the Proteus effect, or actions influenced by social stereotypes, others may show little to no modification in their behavior or perception during SoE (Guy et al., 2023), further highlighting the complexity of SoE.

In terms of assessment methods, the most common method to evaluate SoE is through questionnaires and subjective reports, typically using Likert scales where participants rate their agreement with statements about SoE over a fake body or body part (Guy et al., 2023; Segil et al., 2022). However, variations of these questionnaires are often used across studies, making comparisons difficult and imposing a considerable limitation in the SoE assessment. Efforts to standardize questionnaires, such as the 16-item version by Peck and Gonzalez-Franco (2021), have improved comparability across experiments, but new questionnaires continue to emerge, adding to the complexity (Guy et al., 2023). Furthermore, there are criticisms regarding their reliability due to personal subjectivity, participant interpretation, and scale biases. Therefore, they are not considered the gold standard for SoE assessment despite their usefulness, leading researchers to explore more objective methods.

Behavioral measures like proprioceptive drift, pain perception, intentional binding, and sensory attenuation are often combined with questionnaires for a more comprehensive evaluation.

Proprioceptive drift measures a person's ability to locate a hidden limb without visual feedback. It is calculated as the difference between the actual and perceived positions of the hidden limb after the embodiment illusion, with increased drift indicating a shift in perception toward the fake limb. Thus, it tracks changes in body representation and can be repeated over time to track long-term changes. While linked to ownership illusions, it focuses more on body representation than visuotactile aspects. The SoSL is considered modified when participants point toward the fake limb, suggesting an update in their peripersonal space (Guy et al., 2023; Segil et al., 2022).

Pain perception is based on the hypothesis that disownership reduces pain severity when a painful stimuli are applied to the disowned limb. Pain is typically induced using thermal skin conduction or infrared laser probes, with participants rating pain intensity, and the comparison of pain ratings across conditions gives insight into SoE changes (Segil et al., 2022).

Intentional binding measures time compression between voluntary actions and their sensory outcomes. This phenomenon is closely linked to SoA and is measured by comparing perceived time intervals between self-initiated actions and external events, with greater binding indicates a stronger SoA. However, its reliability is limited by factors like attention and external conditions (Guy et al., 2023; Segil et al., 2022).

Finally, sensory attenuation refers to the decreased perceived intensity of self-generated touch compared to externally generated touch. The brain distinguishes non-threatening self-touch from potentially threatening external touch. Therefore, the attenuation effect indicates whether an individual feels agency over the actions of a fake body part, being again a measure closely related to SoA. It can be tested across multiple sensory modalities, including auditory (e.g., judging sound intensity), visual (e.g., perceiving visual stimuli), and tactile (e.g., applying force to one's own hand and measuring perceived intensity) (Guy et al., 2023; Segil et al., 2022).

While behavioral measures are useful, they alone are insufficient for a complete SoE assessment, and contradictory reports on their reliability further introduce uncertainties regarding these behavioral changes as SoE measurement methods. Thus, physiological measures have been pointed out for a more objective evaluation of SoE. For example, skin temperature, where findings indicate that a decreased skin temperature indicates a feeling of disownership, while increased temperature relates to SoE due to the relationship between skin temperature and autonomic responses. Nonetheless, results are inconsistent, which could stem from uncontrolled factors (e.g., room temperature) (Segil et al., 2022). Similarly, skin conductance measures autonomic responses like sweating. For example, when individuals feel a SoE over an artificial hand, their sympathetic response (such as sweating) is heightened when the hand is threatened. Yet, it is also affected by external factors, such as participant variability and repeated exposure, limiting its reliability over time (Segil et al., 2022). More controlled research is needed to establish these measures as reliable SoE indicators.

Moreover, research into neurophysiological biomarkers using techniques such as Electroencephalography (EEG), Magnetoencephalography (MEG), Functional Magnetic Resonance Imaging (fMRI), Magnetic Resonance Imaging (MRI), Electromyography (EMG), and Near-Infrared Spectroscopy (NIRS) is ongoing, though no definitive conclusions have been reached yet. These non-invasive neuroimaging methods aim to measure brain activity during SoE experiences, to link neurological responses to SoE. This would help to better assess SoE, but also expand the understanding of how one perceives their body and the theoretical framework of embodiment experiences (Segil et al., 2022). Of all techniques, EEG arises as one of the most promising due to its notorious advantages, namely, being a low-cost, portable, and non-invasive procedure that can be applied repeatedly to both adults and children, its high temporal resolution (millisecond level), and the existence of standardized electrode placement and signal processing techniques, tailored to specific application goals, allowing reproducibility and increasing the flexibility of this technique (Teplan, 2022; Gu et al., 2021).

In short, at present, there is no universally accepted objective method for evaluating SoE, and consequently, subjective questionnaires remain the primary tool until more reliable metrics are developed. However, questionnaires are inherently subjective and prone to bias, highlighting the need for robust and objective biomarkers of SoE. Identifying an EEG-based biomarker would provide a more objective means of assessing the illusion, with potential applications in fields such as neurorehabilitation through MI-BCI training and psychological interventions for phobias or trauma. Such a biomarker could enable real-time monitoring to ensure that subjects experience SoE during therapy, facilitating the design of more effective and immersive VR environments. Moreover, physiological assessment methods are essential for advancing our understanding of the underlying neural mechanisms and ensuring consistent evaluation across studies and domains. In particular, EEG-based MI-BCI applications in VR rehabilitation stand to benefit greatly from such developments, motivating the need for a systematic review.

Therefore, the review aims to synthesize EEG findings related to SoE and assess whether consistent EEG changes associated with embodiment have been identified across virtual and non-virtual contexts. It also examines the methodological diversity of existing studies, with the goal of identifying effective approaches and promoting greater consistency and validity in future SoE research. Understanding variability in experimental methods is especially important, as it may significantly influence outcomes (Falcone et al., 2023; Guy et al., 2023).

## 2 Methodology

The Preferred Reporting Items for Systematic Reviews and Meta-Analyses (PRISMA 2020) guidelines (Page et al., 2021) were followed to ensure a rigorous and transparent framework for addressing the research questions (the PRISMA 2020 checklist is provided in the Supplementary material). Selection criteria were defined, and the identified papers were systematically screened for inclusion or exclusion, with the aim of capturing studies reporting EEG-based biomarkers of SoE. The search strategy is described in detail, and the included papers were examined with respect to reported EEG biomarkers, data collection procedures, and methodological approaches.

### 2.1 Inclusion criteria

Articles were included if they explored interventions or exposures related to SoE, such as VR experiences, RHI, MI training, or multisensory integration tasks, while recording and analyzing EEG patterns during illusion. Including and excluding criteria were defined according to the PICOS framework (Table 1). Within this framework, no explicit comparison condition was predefined, as the aim of this review was to identify consistent reports of EEG-based biomarkers associated with SoE across different experimental paradigms, rather than to compare specific control conditions. Studies that did not meet these inclusion criteria, lacked sufficient information, contained redundant data, or did not directly contribute to understanding SoE were excluded from this review.

**Table 1:** Summary of inclusion and exclusion criteria according to the PICOS framework.

**Category**	**Inclusion criteria**	**Exclusion criteria**
Population (P)	Human participants.	Animals or pure simulation studies.
Intervention (I)	Tasks or paradigms designed to explicit induce or modulate SoE, including VR, AR, RHI, MI, and multisensory integration tasks.	Studies not evaluate SoE, or did not explicitly state it.
Comparison (C)	Not applicable (no explicit comparison condition defined).	
Outcome (O)	EEG-derived measures related or accessed during SoE induction.	Studies assessing SoE exclusively through behavioral, subjective, or questionnaire-based measures without EEG.
Study design (S)	Peer-reviewed journal articles and conference proceedings published between 2010 and June 2025, in English.	Reviews, meta-analyses, editorials, theses, non–peer-reviewed articles, or abstracts only. Studies published before 2010, or in non-English language.

### 2.2 Search strategy

The search for an EEG biomarker of SoE was conducted across multiple databases, including ScienceDirect, PubMed, ACM Digital Library, and IEEE Xplore. These databases were selected to ensure comprehensive coverage of the multidisciplinary literature relevant to SoE, EEG, and immersive technologies. ScienceDirect was chosen for its broad coverage of neuroscience and engineering research; PubMed was included to capture studies with biomedical and clinical relevance; the ACM Digital Library was selected to emphasize literature from VR, Human-Computer Interaction (HCI), and game-related research; and IEEE Xplore was included due to its extensive coverage of BCIs, signal processing, and VR/AR technologies.

A common core search strategy was defined and adapted to the specific syntax of each database. The search utilized the following keyword combinations: (“*Sense of Embodiment*” OR “*Body Ownership*”) AND (“*Measure*” OR “*Biomarker*” OR “*Assessment*”), with or without (“*Virtual Reality*” OR “*VR*” OR “*Illusion*”). No exclusion keywords were applied. Filters were applied during this initial search to ensure that the retrieved papers met the predefined inclusion criteria (Table 1). Additionally, reference list screening was not conducted as part of the formal systematic review procedure.

### 2.3 Study selection and data extraction

All screening and eligibility assessments of the included studies were based on their titles and abstracts to determine whether they were relevant to the SoE. Duplicate or not accessible articles were then removed, and the remaining papers were reviewed to ensure they met the inclusion criteria. The second phase of the screening procedure focused on the papers' abstracts, methods, and conclusions to confirm that they focused on modulating SoE and included at least one related physiological measure. Articles that discussed physiological biomarkers of SoE were selected and categorized by the technique used. Then, EEG-related studies were further divided into Augmented Reality (AR), immersive VR, non-immersive VR, and no-VR settings, and were moved to further analysis. Lastly, the EEG-related articles were analyzed in detail, focusing on the methods and results reported.

Similarly, data extraction from these EEG-related studies was performed. The primary outcomes of interest were EEG-derived measures associated with the SoE. As this was an exploratory literature review, no outcome selection process was applied, and all reported EEG outcomes related to SoE were extracted. Additional variables were extracted to examine methodological variability, including sample characteristics, VR paradigms, EEG setups, EEG metrics, and psychometric tools. Due to the exploratory nature of the review and the substantial methodological heterogeneity across experimental paradigms and EEG outcome measures, a narrative synthesis approach was adopted. Studies were grouped by VR modality (AR, immersive VR, non-immersive VR, and no-VR) and EEG feature to facilitate qualitative comparison. Tables were used to report results, supporting their coherent synthesis. Missing or unclear information was not included.

### 2.4 Quality assessment

Study quality and risk of bias were assessed using the Joanna Briggs Institute (JBI) Critical Appraisal Checklist for Analytical Cross-Sectional Studies (Moola, 2024), a method that evaluates methodological quality and potential sources of bias in study design, conduct, and analysis. Although originally developed for clinical and epidemiological research, key appraisal domains apply to the experimental studies included in this review, to provide a structured, transparent approach to assessing methodological rigor across heterogeneous study designs. The first author assessed each study according to the eight questions from the JBI checklist that covered participant selection, confounders, outcome measurement, and statistical analysis. For the purposes of this review, the JBI checklist items were interpreted as it follows: Q1 (“*Were the criteria for inclusion in the sample clearly defined?*”) was interpreted as the explicit definition of inclusion/exclusion criteria of participants beyond vague descriptions; Q2 (“*Were the study subjects and the setting described in detail?*”) was assessed based on whether the sample was adequately described in terms of demographic characteristics (e.g., age, sex/gender, and sample size); Q3 (“*Was the exposure measured in a valid and reliable way?*”) was interpreted in relation to whether the experimental conditions were clearly defined and appropriately implemented to manipulate SoE; Q4 (“*Were objective, standard criteria used for measurement of the condition?*”) was evaluated based on whether the authors clearly defined what aspects of SoE were being assessed and how these were measured, using standardized methods; Q5 (“*Were confounding factors identified?*”) focused on whether potential confounders related to study design, SoE implementation, or EEG analysis, were acknowledged; Q6 (“*Were strategies to deal with confounding factors stated?*”) was assessed based on whether at least some of the identified confounders were addressed or explicitly reported as study limitations; Q7 (“*Were the outcomes measured in a valid and reliable way?*”) was interpreted in the context of EEG recording, preprocessing, analysis, and outcome definition; and Q8 (“*Was appropriate statistical analysis used?*”) was assessed based on whether the statistical analyses were suitable for the study design and were adequately reported. The responses were scored as “*yes*,” “*no*,” “*unclear*,” or “not applicable,” and the results were summarized descriptively to characterize common sources of bias across studies.

## 3 Results

After the search, 602 papers were initially identified across the databases. After removing duplicates and excluding articles without access, 478 studies remained for further analysis to determine if they met the inclusion criteria. Ultimately, 134 papers were found to meet the criteria, addressing SoE biomarkers. Of these, 20 were reviews, 80 focused on different physiological techniques (such as skin temperature, EMG, MEG, fMRI, or MRI, skin conductance, cardiac and blood perfusion, eye tracking, NIRS, enzyme and molecular studies, physiological activity in deep visceral organs, etc.), and 35 focused on EEG. Overall, EEG was the most common physiological technique for identifying a SoE biomarker in the last fifteen years.

**Figure 1:**
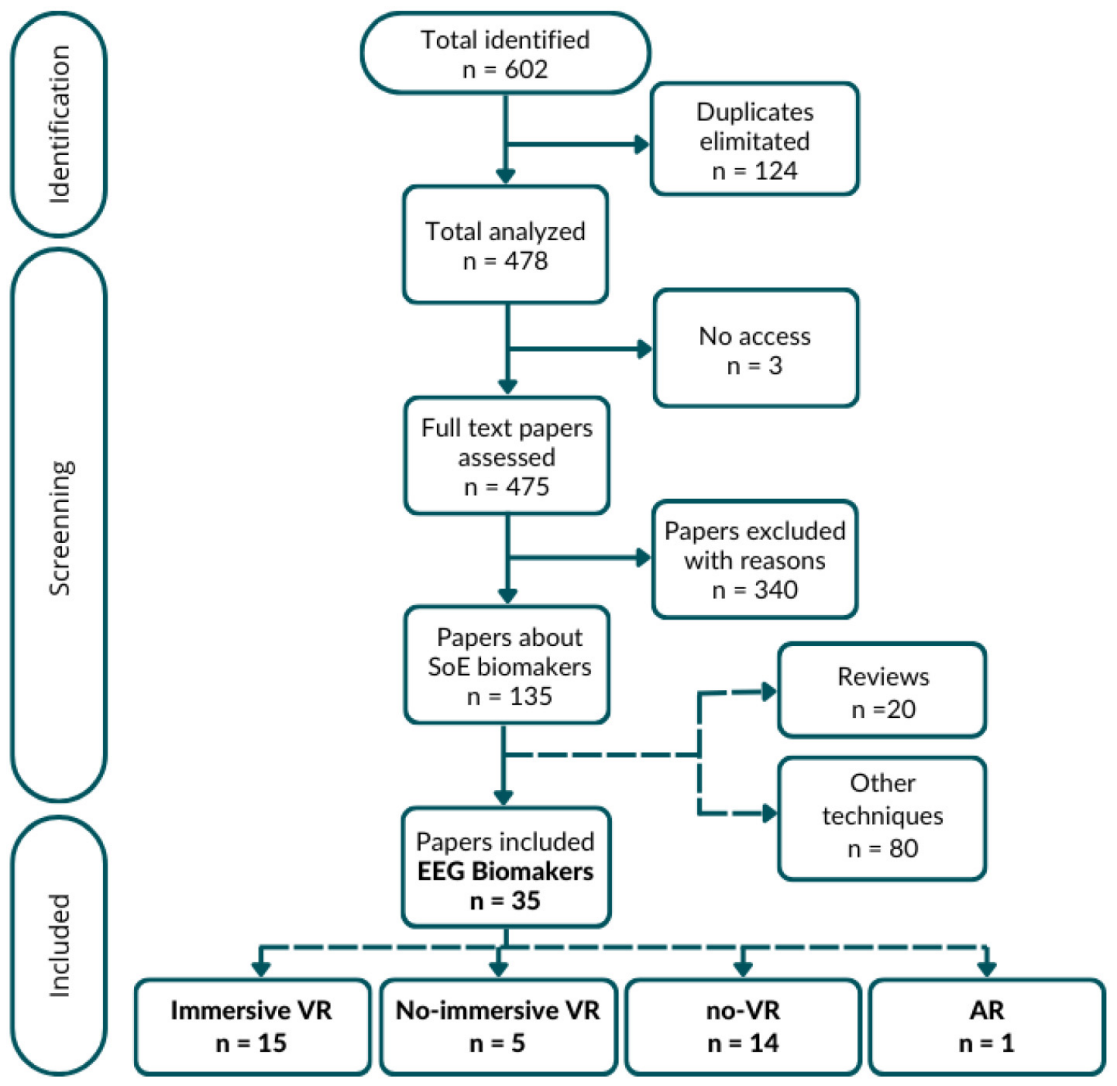
Flowchart illustrating the identification, screening, and selection process of studies included in the analysis of EEG biomarkers associated with the SoE.

In the end, those 35 articles specifically related to EEG biomarkers of SoE were included for analysis. These articles were further categorized into AR (1 article), immersive VR (15 articles), non-immersive VR (5 articles), and no-VR settings (14 articles). The screening process for the selected articles is illustrated in Figure 1.

**Figure 2:**
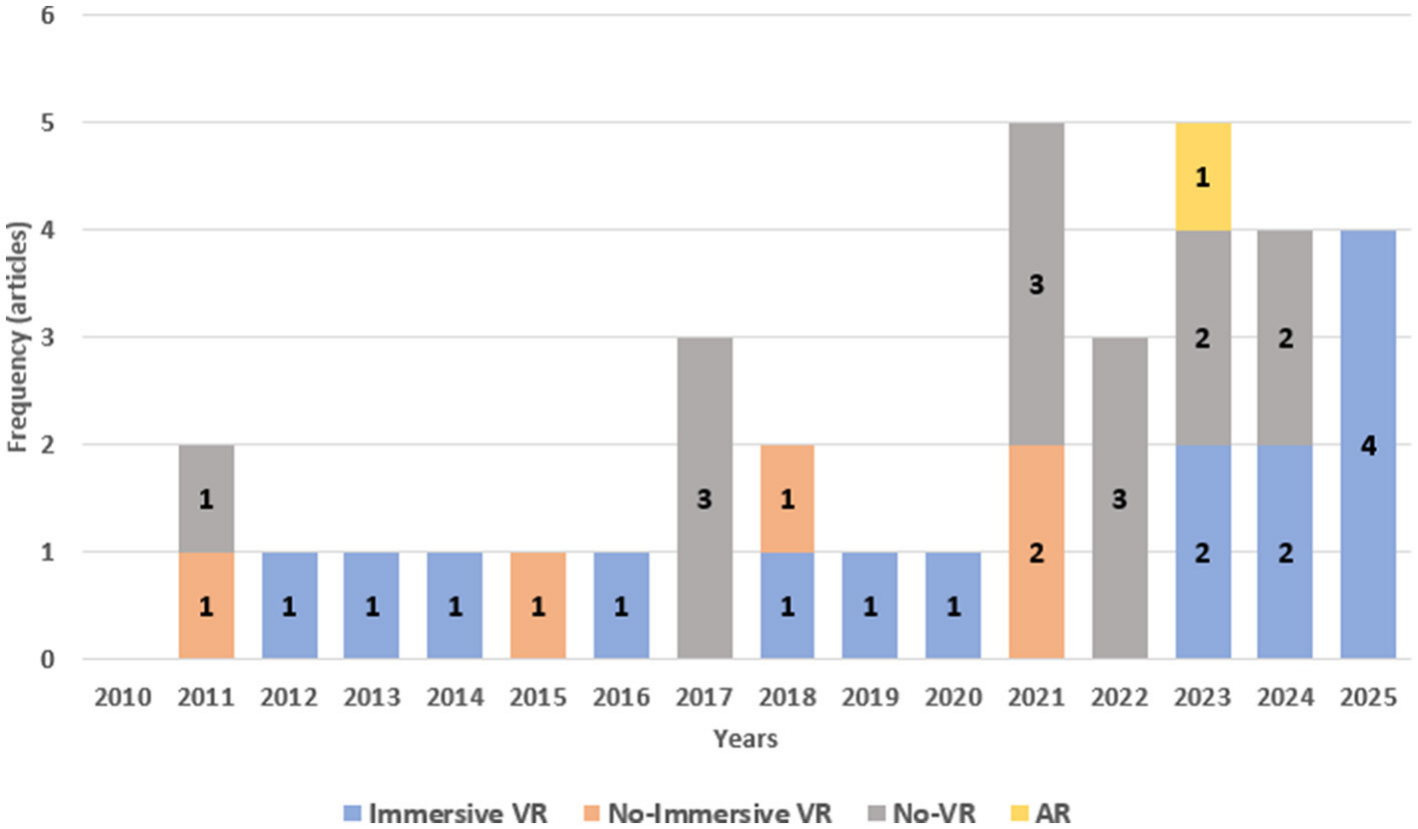
Histogram showing the number of articles included in the analysis of EEG biomarkers associated with the SoE, categorized by year. Blue bars represent studies using immersive VR to induce the illusion, orange bars represent non-immersive VR settings, gray bars represent studies using other methods to induce the illusion (no-VR settings), and, lastly, the yellow bar represents the study using AR.

Figure 2 presents a histogram showing the number of articles included in this review per year. An increase in studies was expected due to the growing interest in SoE across various fields, particularly in restorative MI-BCI research. This trend was further driven by the rapid adoption of VR, especially following recent developments in HMD that have made immersive VR more accessible and user-friendly. Consequently, an increase in SoE-focused studies using VR settings was anticipated. As shown in the histogram, the number of studies indeed grew over time.

### 3.1 EEG biomarkers of SoE

Table 2 summarizes the most notable findings from the included papers, highlighting conclusions regarding potential EEG biomarkers of SoE. Overall, the literature indicates that embodiment illusions can modulate EEG signals, with several potential biomarkers reported across different frequency bands. However, there is also a considerable amount of conflicting evidence.

**Table 2:** Summary of the most relevant findings reported in the articles included in the literature review.

**Category**		**Findings**	**Articles**			
			**Immersive VR**	**No immersive VR**	**No-VR**	**AR**
Oscillatory Processes		There is an increase of mid-frontal theta power in response to observing avatar errors when embodied.	(Pavone et al., 2016)			
		There is a stronger theta ERS in the left frontocentral areas during high levels of SoA in pre- movement of the hand, while this increase is observed in the right temporal areas during low SoA during hand movement.	(Jeunet et al., 2018)			
	Theta Band	SoE is related to theta power modulation in the central region.	(Esteves et al., 2025), (Ramírez-Campos et al., 2024)			
		Greater theta bands activity in the temporal and adjacent areas occurs during incongruent visuotactile stimuli (low SoE), indicating a greater workload to assimilate the incongruent inputs.				(Hansford et al., 2023)
		No changes in theta power during SoE.	(Li et al., 2023; Raz et al., 2020)	(Sciortino and Kayser, 2022b)		
		SoE is related to alpha activity over central–parietal regions.	(Ramírez-Campos et al., 2024)			
		SoE is associated with stronger alpha band suppression in the frontal, parietal, and central regions, mainly in the sensorimotor cortex.	(Raz et al., 2020; Nicolardi et al., 2025)		(Kang et al., 2015), (Shibuya et al., 2021), (Shibuya et al., 2018) (Sciortino and Kayser, 2022b), (Faivre et al., 2017), (Rao and Kayser, 2017), (Della Longa et al., 2021), (Shibuya and Ohki, 2023)	
		When mentally or physically controlling an avatar's gait, SoE is associated with higher alpha ERS in centrofrontal and centro-parietal areas.	(Alchalabi et al., 2019)			
	Alpha Band	Higher frontal alpha power is associated with decreased susceptibility to SoE illusions.		(Hsu et al., 2022), (Della Longa et al., 2021)		
		SoSL is associated with a stronger alpha band over the mPFC. There is less alpha suppression over fronto-parietal areas during SoO illusions.	(Evans and Blanke, 2013)		(Lenggenhager et al., 2011)	
		There is an increase of alpha band desynchronization over parietal areas in response to observing avatar errors when embodied.	(Pavone et al., 2016)			
		No lateralization of alpha power reduction was observed during SoE.		(Sciortino and Kayser, 2022b)		
		No changes in alpha band during SoE.	(Li et al., 2023; Esteves et al., 2025)			(Hansford et al., 2023)
		SoE is associated with beta power increases in the occipital region.	(Esteves et al., 2025)			
		SoE is associated with a decrease of beta power over frontal, central, and parietal, mainly sensorimotor brain areas.	(Kang et al., 2015)	(Sciortino and Kayser, 2022b), (Rao and Kayser, 2017), (Faivre et al., 2017)		
		No lateralization of beta power reduction during SoE.		(Sciortino and Kayser, 2022b)		
	Beta Band	Beta power reduction during RHI (SoE) emerges immediately after the illusion onset.		(Sciortino and Kayser, 2022b)		
		SoO is associated with attenuated beta ERD over contralateral sensorimotor brain areas.			(Shibuya et al., 2021)	
		Behavioral SoE measure (proprioceptive drift) is associated with stronger high beta power over fronto- temporal sites.		(Faivre et al., 2017)		
		No changes in beta power during SoE.	(Alchalabi et al., 2019; Li et al., 2023; Evans and Blanke, 2013; Raz et al., 2020)			(Hansford et al., 2023)
		SoE is associated to gamma activity over central–parietal regions.	(Ramírez-Campos et al., 2024)			(Hansford et al., 2023)
		SoE is associated with increased gamma activity in frontal, central, and parietal regions, particularly in the somatosensory cortex.	(Li et al., 2023; Esteves et al., 2025)	(Hiramoto et al., 2017)		(Hansford et al., 2023)
	Gamma Band	SoE is associated with gamma power increases in the occipital regions.	(Esteves et al., 2025)			
		Decreased gamma band activity over central and parietal areas does not directly correlate with the SoA, but may be related to general visuomotor processing.	(Kang et al., 2015)			
		SoE is associated with increased gamma-band connectivity between sensorimotor and occipital/visual regions, particularly during illusory self-touch conditions.	(Li et al., 2023)	(Faivre et al., 2017)		
		No changes in gamma power during SoE.	(Alchalabi et al., 2019; Raz et al., 2020; Evans and Blanke, 2013)		(Lenggenhager et al., 2011)	(Sciortino and Kayser, 2022b)
		Individuals less susceptible to the illusion have higher communication across delta and alpha bands, with delta modulating alpha power over frontal areas.		(Hsu et al., 2022)		
	Multi-band dynamics	There may be an association between beta-to-alpha wave ratio and SoA and SoO over the occipital lobe.		(Shimizu et al., 2021)		
		SoO is associated with PSD increases in associative scalp regions, including the frontal, parietal, and central areas.		(Blefari et al., 2011)		
		Different EEG signatures occur in early vs. late neural activity depending on SoA presence.		(Veillette et al., 2023)		
	SEP	SoE is associated with enhanced SEP, particularly the early P40 component, suggesting modulation of activity in the primary somatosensory cortex. It is also associated with lower amplitude and shorter duration in the later SEP response (110–200 ms) at a higher-tier somatosensory region.	(Aspell et al., 2012)			
		There is an attenuation of the N1-P1 component during RHI (high SoO), in the primary somatosensory cortex.		(Sakamoto and Ifuku, 2021)		
		SoO is associated with motor cortex activation, specifically increased P450 amplitude, when threatening the embodied hand.	(Gonzälez-Franco et al., 2014)			
		SoE is associated with a smaller N2 (200–250 ms) amplitude in the frontal brain area.	(Lu et al., 2025)			
		SoE is associated with an early (~150 ms) frontocentral ERP positivity, localized to the SMA and midcingulate cortex.		(Heldmann et al., 2024)		
	Other ERP	The N270 component increases in response to high body self-identification in the frontal and central areas.				
Evoked Potentials		SoE is associated with a weaker N450 wave over the central lobe when receiving somatosensorial cues.			(Sebastiano et al., 2024)	
		SoE is associated with the attenuation of ERP around 330 ms over frontocentral regions.		(Rao and Kayser, 2017)		
		Evoked responses to the RHI (high SoO) create a specific neurophysiological signature, especially dominant around 125ms-275ms following stimulus onset.		(Sciortino and Kayser, 2022a)		
	ErrPs	Prior embodiment modulates ErrPs. In its absence, ERN and Pe amplitudes increase, and an N400 wave emerges over fronto-central areas during the observation of avatar errors.	(Porssut et al., 2023)			
	(ERN, Pe, and N400)	ErrPs over central and parietal regions may serve as a marker of breaks in embodiment.	(Iwane et al., 2024)			
		SoO is associated with reduced Pe wave.	(Raz et al., 2020)			
		SoE is associated with stronger ERN and N400 responses when observing avatar errors, while Pe remained unaffected.	(Pavone et al., 2016)			
Fractal dimension		SoA is associated with a higher fractal dimension of the EEG signal measured across nearly the entire scalp.		(Veillette et al., 2023)		
Other signals		SoE illusions enhance muscle excitability, and signal complexity, indicating robust neural and muscular engagement.	(Li et al., 2023)			

#### 3.1.1 Oscillatory processes

Power changes across frequency bands: Delta (1–4 Hz), Theta (4–7 Hz), Alpha (8–12 Hz), Beta (13–30 Hz), and Gamma (>30 Hz; including low- and high-gamma ranges), were the most commonly investigated features for potential biomarkers. However, only three articles using VR settings included the Delta band in this power spectral analysis, and those that did found any relationship between Delta and SoE (Jeunet et al., 2018; Li et al., 2023; Hansford et al., 2023). Esteves et al. (2025) reported minor occipital delta decreases during the illusion, but without significant differences between embodiment and control conditions. Similarly, only eight studies examined Theta power in relation to SoE. Two found correlations between SoE and Theta modulation in central regions (Ramírez-Campos et al., 2024; Esteves et al., 2025), while another reported increased Theta activity in temporal regions during incongruent visuotactile stimuli (no SoE), suggesting higher cognitive effort to resolve conflicting inputs (Hansford et al., 2023). Moreover, Jeunet et al. (2018) observed stronger Theta ERS in left frontocentral areas during a high SoA prior to movement onset, whereas low SoA was associated with Theta increases in right temporal regions. Although these findings relate to SoA specifically, they suggest that Theta activity may vary depending on the presence or absence of embodiment illusions. Nonetheless, three other studies (one no-VR and two in VR settings) reported no changes in the Theta band associated with the SoE illusion (Raz et al., 2020; Li et al., 2023; Sciortino and Kayser, 2022b), making it difficult to draw a definitive conclusion.

This highlights a substantial gap in understanding the relationship between low-frequency bands, particularly Delta and Theta, and SoE. In contrast, Beta and Gamma rhythms have been more widely studied during embodiment illusions, yet results remain mixed.

Among these, the Beta band has received particular attention, with eleven studies analyzing its power. Four of these reported Beta power decreases over sensorimotor areas correlated with stronger SoA (Kang et al., 2015; Sciortino and Kayser, 2022b; Rao and Kayser, 2017; Faivre et al., 2017). In contrast, Shibuya et al. (2021) observed weaker Beta-ERD (i.e., higher Beta power) over contralateral sensorimotor areas when participants observed rotation of an embodied fake hand compared to a disembodied hand. Similarly, Esteves et al. (2025) reported increased Beta power over occipital regions during the SoE illusion relative to disembodied conditions. These divergent findings suggest that Beta modulation may be sensitive to experimental paradigms, brain regions analyzed, and the type of sensorimotor cues.

For Gamma power, some studies also reported its modulation during SoE (Ramírez-Campos et al., 2024; Hansford et al., 2023), specifically, increased activity in frontal, central, parietal, and/or occipital regions (Li et al., 2023; Hansford et al., 2023; Esteves et al., 2025), or enhanced connectivity between sensorimotor and occipital areas (Li et al., 2023; Faivre et al., 2017), emphasizing the link between visual feedback and the illusion. These changes indicate enhanced cortical excitability and inter-regional communication, suggesting that Gamma connectivity may serve as a neural marker of embodiment. Nonetheless, Kang et al. (2015) reported a weak Gamma decrease during high SoA, although this was not correlated with SoA scores. The authors suggested that the Gamma changes are probably related to general visuomotor processing, which can explain the different findings. This observation opens more questions regarding the complexity between SoE and its components and how that affects brain activity. Furthermore, Hiramoto et al. (2017) reported reduced low-Gamma and increased high-Gamma power during congruent visuotactile stimulation (RHI) compared to incongruent controls, implying that different Gamma sub-bands may associate with SoE in distinct ways.

In summary, the most consistent findings point to increased Gamma-band activity, while Beta power often decreases during embodiment illusions. However, five studies reported no significant differences in Beta power (Raz et al., 2020; Li et al., 2023; Evans and Blanke, 2013; Alchalabi et al., 2019; Hansford et al., 2023), and four studies did not find changes in Gamma power (Raz et al., 2020; Li et al., 2023; Evans and Blanke, 2013; Alchalabi et al., 2019; Lenggenhager et al., 2011) during illusion in VR/AR settings. This pattern extends to non-VR contexts, with one study also reporting no differences in Gamma power (Sciortino and Kayser, 2022b). Overall, contradictory results appear more frequently in VR studies, highlighting the possible influence of experimental paradigms and feedback delivery methods on EEG patterns during embodiment illusions.

The most consistently reported EEG change in the literature is a greater decrease in Alpha power, particularly over central and parietal regions, which correlates with strong SoE. This has been observed in two immersive VR studies (Raz et al., 2020; Nicolardi et al., 2025), three non-immersive VR studies (Kang et al., 2015; Shibuya et al., 2021, 2018), and five non-VR studies (Sciortino and Kayser, 2022b; Faivre et al., 2017; Rao and Kayser, 2017; Della Longa et al., 2021; Shibuya and Ohki, 2023), making it the most reliable EEG indicator of embodiment illusions. This effect primarily occurs over somatosensory areas and appears largely non-lateralized (Sciortino and Kayser, 2022b). Nevertheless, some studies report exceptions. For example, Evans and Blanke (2013) found reduced Alpha suppression over fronto-parietal areas during embodiment, while Lenggenhager et al. (2011) observed Alpha activation in the medial prefrontal cortex (mPFC) associated with SoSL. Similarly, Alchalabi et al. (2019) suggested that Alpha ERS over centro-frontal and centro-parietal regions could be leveraged to control avatar gait. However, other two VR studies were unable to detect Alpha changes (Li et al., 2023; Esteves et al., 2025), preventing Alpha from being a universal neural signature of SoE.

Additionally, Hsu et al. (2022) reported that increased frontal Alpha power, particularly during eyes-closed resting state, is associated with lower susceptibility to ownership illusions (e.g., RHI). This study further suggested that Delta activity modulates frontal Alpha power in low-susceptibility participants, highlighting possible cross-frequency interactions. Supporting this observation, Della Longa et al. (2021) indicates that weaker frontal Alpha activation is associated with (preterm and full-term) children more susceptible to the illusion.

Finally, still related to oscillatory processes, Blefari et al. (2011) found SoO to be associated with general power spectral density (PSD) increases in frontal, parietal, and central regions, whereas Shimizu et al. (2021) attempted to discriminate SoO and SoA using Beta-to-Alpha ratios, showing some association between this occipital wave ratio and SoA and SoO. Although some associations were observed, the small sample size and highly localized effect limited their reliability. Temporal analyses further enrich this picture. One study showed distinct early and late neural processes depending on SoA presence, while also finding that EEG fractal complexity (fractal dimension) increases during self-attributed movements (Veillette et al., 2023), suggesting that neural complexity may represent a novel marker of agency. Similarly, one VR study found that threatening an embodied virtual hand elicited increased P450 amplitude, mirroring real-life threat responses (Gonzälez-Franco et al., 2014).

#### 3.1.2 Evoked potentials

Changes in evoked potentials have also being reported in the literature, namely, Event-Related Potentials (ERP), Somatosensory Evoked Potentials (SEP), and Error-Related Potentials (ErrPs).

ERP components linked to SoE in other contexts in at least six studies. For example, Lu et al. (2025) found that stronger self-identity and higher SoE were associated with smaller N2 amplitudes (200–250 ms) over the frontal lobe, suggesting automatic self-recognition. They also reported P1 changes (80–130 ms) over occipital regions, mainly reflecting body size processing, and N170 modulations (140–190 ms) over temporal areas, linked to face recognition. Importantly, participants embodied more easily in self-avatars, with both body and face images contributing equally to self-recognition. On this line of self-recognition tasks, Galigani et al. (2021) observed increased N270 responses when participants identified their own body, linking this component to body ownership. Similarly, Sciortino and Kayser (2022a) applied linear discriminant analysis to evoked responses and showed reliable distinctions between illusion and non-illusion conditions, with earliest differences emerging around 65 ms and a dominant neurophysiological signature between 125–275 ms. During the RHI, Rao and Kayser (2017) reported a negative ERP deflection at ~330 ms over frontocentral regions (FCz), consistently associated with subjective illusion experience. However, some effects appeared only in specific control contrasts, highlighting the importance of careful experimental design. Sebastiano et al. (2024) further noted modality-specific differences by showing that RHI reduces somatosensory-evoked responses but enhances visual-evoked responses. In particular, weaker N450 and P300 waves were observed over central and frontal areas with somatosensory cues, while stronger P2–N3 and N2 responses emerged over central and occipital regions with visual cues. Of these, only the reduced N450 over the central lobe was directly correlated with embodiment.

Lastly, evidence from non-VR paradigms also supports early ERP markers of embodiment. Heldmann et al. (2024) showed that the embodiment illusion elicited greater frontocentral ERP positivity between 100–200 ms, especially in response to non-threatening brush strokes compared to threatening needle pricks. Source localization suggested predominant activity in the supplementary motor area (SMA) and midcingulate cortex, indicating a possible SoE biomarker: an early (~150 ms) frontocentral positivity localized to SMA/midcingulate regions.

Regarding SEPs, two studies reported associations with embodiment. For example, illusion-based self-identification in VR modulates somatosensory cortex activity at different latencies, particularly enhancing the early P40 component, while later SEP responses (110–200 ms) in higher-tier somatosensory regions show reduced amplitude and shorter duration (Aspell et al., 2012). Similarly, during the RHI (high SoO), an attenuation of the N1–P1 component has been observed in the primary somatosensory cortex (Sakamoto and Ifuku, 2021).

Finally, ErrPs associated with SoE comprise the ERN, Pe, and N400 components. For instance, Porssut et al. (2023) showed that prior embodiment in VR modulates brain responses to avatar errors, specifically, in the absence of embodiment, ERN and Pe amplitudes increased, and an N400 wave emerged over frontocentral regions. Consistently, reduced Pe amplitudes during embodiment have also been reported as a correlate of SoO (Raz et al., 2020). In contrast, Pavone et al. (2016) found that observing errors in an embodied avatar elicited stronger ERN and N400 responses, while Pe remained unaffected. These error-related effects were accompanied by increased mid-frontal Theta power and greater Alpha desynchronization over parietal regions, suggesting that embodiment primarily modulates early, automatic stages of error processing. Finally, Iwane et al. (2024) demonstrated that breaks in SoE can be detected through ErrPs, with distortions in an avatar's movement during embodiment eliciting a distinct EEG sequence of negative–positive–negative deflections over central and parietal regions, which only occurred when participants were previously embodied in the avatar.

Overall, while several EEG features like oscillatory processes, or evoked potentials have been linked to embodiment illusions, findings remain fragmented and inconsistent. Across studies, the most congruent observation is not a single biomarker, but rather the complexity of SoE itself and its dependence on paradigm design and feedback modalities.

### 3.2 Paradigms and data collection methods implemented

As most of the studies included in this review emphasize, the type of feedback provided during the embodiment illusion directly impacts EEG activity and how participants process the illusion. For this reason, understanding the paradigm and methodological choices of each study is crucial. However, as summarized in Table 3, the included studies differ widely in their design, introducing substantial variability that may partly explain the heterogeneity of reported results.

**Table 3:** Overview of the experimental paradigms and methodological characteristics of the EEG studies, grouped by immersive VR, non-immersive VR, no-VR, and AR settings.

**Paradigm**	**Articles**	**Number of participants**	**EEG setup**	**EEG processing**	**SoE induction triggers**	**SoE mesurement**	**Paradigma**
Immersive VR settings	(Gonzälez-Franco et al., 2014)	19 healthy, right-handed participatns	6 electrodes (10–20 system)	Not stated; time and time-frequency analyses	Visuoproprioceptive. Looking around the VE	2 questions related to SoO (5-point Likert scale)	Within-subject; VR coherent with the real world; Stitted on a chair, with the right hand on the table in front of them, condtioins consited of appearing a knife than stabed the virtual hand or the table.
	(Jeunet et al., 2018)	24 naive, right-handed participatns	32 electrodes (10–20 system)	Clearly stated; time- frequency analysis	Visuoproprioceptive and Visuomotor	1 question related to SoA (7-point Likert scale)	Within-subject; VR scenario distinct from the real world. Participants were seated in the virtual world, facing a TV screen where one of three different hand movements was displayed. After viewing the movement, participants were instructed to reproduce it using their own hands. Each movement was present under 4 conditions: one non-manipulated (with correct visuomotor synchrony) and three manipulated (designed to disrupt agency).
	(Aspell et al., 2012)	18 healthy, right-handed participants	32 electrodes (10–20 system)	Clearly stated; time- locked analysis	Visuoproprioceptive (real scenario in 3PP) and Visuotactile	8 questions related to SoE (5-point Likert scale)	Within-subject design; VR scenario replicating the real world (via a live camera feed projected into the VR headset). Participants were seated and focused on their virtual back, where four LEDs were displayed. They received tactile vibrations on their back while ignoring distracting LED flashes, and had to press a button as quickly as possible to indicate whether the vibration occurred on the upper or lower part of their back. There were four experimental conditions, based on a 2 × 2 design: Temporal alignment: Synchronous vs. Asynchronous LED flashing relative to the vibration; and Spatial alignment: Same side vs. Opposite side of the back.
	(Alchalabi et al., 2019)	20 healthy, naive participants	19 electrodes (10–20 system)	Clearly stated; frequen- cy analysis	Visuoproprioceptive and Visuomotor	9 questions related to SoE (7-point Likert scale)	Within-subject design; VR scenario replicating a real hallway. Participants stood on a treadmill and viewed a virtual self-avatar, focusing on their lower limbs. In each trial, an arrow appeared, pointing left, right, or forward. Based on this cue, participants had to either physically perform the movement (GO), imagine (IMAGINE), or observe it (OBSERVE). The task was presented with two types of visual feedback: congruent or incongruent.
	(Porssut et al., 2023)	19 healthy, right-handed participants	32 electrodes (10–10 system)	Clearly stated; time- locked analysis	Visuoproprioceptive and Visuomotor	1 question related to SoO (rated on a visual color gradient scale from 0 to 100)	Within-subject design; VR scenario replicating the real world. It began with a SoE induction, where participants did reaching tasks. Then, participants performed the main task following a 2 × 2 design. First, they were presented with either the Embodiment or No-Embodiment condition (participants rotated their hand and either saw the virtual hand mimic the movement or not, respectively). Then, they experienced either a Disruption or No-Disruption condition (they were instructed to remain still and focus on the virtual hand, which either moved unexpectedly or remained still, respectively).
	(Raz et al., 2020)	18 healthy participants	12 electrodes (10–20 system)	Clearly stated; time and time-frequency analyses	Visuoproprioceptive, Visuomotor and Visuotactile	Proprioceptive drift, and 4 questions related to SoO (7-point Likert scale)	Within-subject design; VR scenario mimicking the real world. Participants completed five VR sessions manipulating: agency (active/passive), synchronicity (synchronous/asynchronous), and semantic congruence (realistic/cursor/mirrored hands). Each trial included: hand position estimation (proprioceptive drift), ownership induction via palm sliding (realistic/cursor/mirrored hands) using visuomotor cues or via stroking (synchronous/asynchronous) using visuotactile cues, and a hands-attending task, where participants viewed unexpected virtual hand (realistic/cursor/mirrored hands) movements.
	(Pavone et al., 2016)	20 healthy, naive and right-handed participants	60 electrodes (10–10 system)	Clearly stated; time and time-frequency analyses	Visuoproprioceptive	1 question related to SoE (5-point Likert scale)	Within-subject design; VR scenario different from the real world. Participants sat at a table with two mugs in front of them, viewing one avatar from a 1PP and another from a 3PP. There were two conditions: in one, the 1PP avatar performed grasping movements toward the mugs; in the other, the 3PP avatar did, while participants observed the movements. In 70% of the trials, the grasping movement was correct, while in 30%, it missed the mug (error).
	(Li et al., 2023)	35 healthy, right-handed participants	39 electrodes (10–20 system)	Clearly stated; frequency, connectivity, and comple- xity analyses	Visuoproprioceptive	1 question related to SoO, and 1 question related to kinesthetic illusion (7-point Likert scale)	Within-subject design; VR using pre-recorded videos. Participants sat in a relaxed position while viewing videos of their own legs performing seated front kicks (left/right, 30°/60°). Two conditions were presented: an illusion condition, where the video was spatially aligned, and an observation condition, where the same video was shown without alignment on a screen, allowing simultaneous viewing of both the real and virtual leg.
	(Evans and Blanke, 2013)	12 healthy, right-handed participants	64 electrodes (10–20 system)	Clearly stated; frequency analysis and source locali- zation	Visuoproprioceptive and Visuotactile	4 questions related to SoO (7-point Likert scale)	Within-subject design; VR closely matched to the real world. Participants viewed either virtual arms or non-body objects resting on a virtual table, while tactile feedback was delivered via vibration motors attached to both hands. The experiment followed a 2 × 2 design: Stroking (synchronous vs. asynchronous visuo-tactile stimulation) and Object (virtual arms vs. control objects). Additionally, a baseline condition with no stimulation and a MI task were included, in which participants imagined clasping movements with either hand while viewing the same VR scene.
	(Iwane et al., 2024)	37 healthy participants	32 electrodes (10–10 system)	Clearly stated; time-locked analysis	Visuoproprioceptive and Visuomotor	2 questions of “yes” or “no”	Within-subject design; VR was a room with a chair. Participants wore an HTC Vive Pro Eye HMD with motion and eye tracking, and haptic feedback via a real–virtual tennis ball alignment. Three groups completed phases of calibration, explanation, practice, and distortion-adaptation (first two groups also had a decoder-calibration phase for personalized error-related potential decoders). Each trial involved three reaching movements toward moving targets with varying visuomotor distortions, followed by two yes/no questions on distortion perception and body ownership. Distortion strength was adjusted to optimize avatar–human mapping using either reinforcement learning or a non-personalized decoder, depending on the group.
	(Ramírez-Campos et al., 2024)	26 healthy, right-handed participants	32 electrodes (10–20 system)	Clearly stated; machine- learning analysis	Visuoproprioceptive, Visuomotor and Visuotactile	16 questions related to SoE (7-point Likert scale)	Public dataset with a between-subject design; the virtual environment mirrored the real world. The procedure included two phases: (1) a 5-min embodiment priming - experiment group explored the environment from a first-person perspective with visuomotor coherence for 3 min followed by 2 min of VHI, while the control group received asynchronous third-person cues; and (2) a 10-min MI training in a similar virtual setup (40 trials of left/right hand fist imagery with rest periods).
	(Esteves et al., 2025)	41 healthy, right-handed participants	32 electrodes (10–20 system)	Clearly stated; frequency analysis	Visuoproprioceptive, Visuomotor and Visuotactile	16 questions related to SoE (7-point Likert scale)	26 participants (between-subject) and 15 participants (within-subject); the virtual environment mirrored the real world. Participants experienced an embodied condition (5 min: 3 min first-person exploratiowith visuomotor coherence + 2 min VHI) and a control condition (5 min: 3 min third-person exploration with asynchronous cues + 2 min incorrect VHI).
	(Nicolardi et al., 2025)	24 healthy, right-handed participants	64 electrodes (10–10 system)	Clearly stated; time and time-frequency analyses	Visuoproprioceptive and Visuotactile	3 questions related to SoO, rated on a continuous scale of 60cm	Within-subject design; participants sat at a table in VR with their right hand positioned above it. They completed 90 first-person and 90 third-person trials (equally divided among painful, pleasant, and neutral stimuli, presented randomly). Each trial included hand observation, a visuotactile stimulus, and a final hand observation, followed by embodiment questions.
	(Lu et al., 2025)	29 healthy, female participants	64 electrodes (10–20 system)	Clearly stated; time analysis	Visuoproprioceptive and Visuomotor	3 questions related to SoE (7-point Likert scale)	Within-subject design; Participants completed four conditions (self/other face × normal/altered body), each with three phases: (1) Embodiment induction in VR via a bubble-catching task; (2) EEG recording, while viewing avatar images (3 shirt colors × 3 orientations) and performed an oddball detection task (pressing a button for deviant avatars; 378 standard, 36 deviant trials); (3) Post-questionnaire.
	(Miremadi et al., 2025)	25 healthy participants	24 electrodes (10–20 system)	Clearly stated; frequency analysis	Visuoproprioceptive and Visuomotor	Implicitly assumed based on the tasks (without direct measurement)	Within-subject design; participants completed a 20-minute VR-based upper limb training program (KarunaHOME) consisting of five motor tasks (Calibration, Lotus Toss, Connect the Dots, Mirroring, Starry Night) presented in random order. The tasks alternated between simple and complex arm movements and were interspersed with guided breathing exercises. Movements involved the dominant, non-dominant, or mirrored arm.
No immersive VR settings	(Lenggenhager et al., 2011)	11 healthy, right-handed participants	256 electrodes (radial layout)	Clearly stated; frequency analysis	Visuoproprioceptive and Visuotactile	Proprioceptive drift, and pressing a button when feedback was conguent	Within-subject; 3D room with projection on a real-size screen; Participants stood in front of the screen, where they saw an avatar (humanoid or object). Participants were stroked on their back using a motion-tracked stick, while seeing the virtual object being touched synchronously or asynchronously (4 conditions, Object or Humanoid × Synchronous or Asynchronous touch).
	(Kang et al., 2015)	19 healthy, naive, and right-handed participants	19 electrodes analyised (10–20 system)	Clearly stated; frequency and connectivity analyses	Visuomotor	1 question related to the feeling of control over the virtual hand (0 to 100 scale)	Within-subject design; computer screen. Participants wore a glove on their right hand to control a virtual hand displayed on a monitor. They could see the virtual hand but not their real hand. During the initial phase of each trial, they were instructed to fixate on a cross on the screen and remain still. When the cross disappeared, they were allowed to move their right hand freely, with the virtual hand visually imitating their movements. The level of visual feedback modulation varied across trials: 0%, 25%, 50%, 75%, or 100% control over the movements of the virtual hand.
	(Shibuya et al., 2021)	24 healthy participants	32 electrodes (10–20 system)	Clearly stated; time- frequency analysis	Visuoproprioceptive and Visuotactile	Proprioceptive drift, and 9 questions related to SoO and SoA (7-point Likert scale)	Within-subject design; RHI of the left hand using a fake hand projected on a screen. Participants underwent four experimental conditions in a randomized order, based on a 2 × 2 factorial design: synchrony (synchronous vs. asynchronous stimulation) and rotation (medial vs. lateral). Each of the 36 trials per condition began with synchronous or asynchronous brush strokes, followed by a 30° rotation of the fake hand (clockwise for the medial condition), and counterclockwise for the lateral condition.
	(Galigani et al., 2021)	15 healthy, right-handed participants	32 electrodes (10–20 system)	Clearly stated; time analysis	Visual self-recognition	Correct identification of self-hand and response time	Within-subject design; Computer screen. Participants viewed pairs of standardized grayscale images of the dorsum of right hands on a computer screen and judged whether each pair was identical or different. Stimuli were briefly presented with jittered inter-pair intervals to prevent anticipation. Images appeared in three scenarios: With Self (including the participant's own hand and a stranger's), Without Self (only unfamiliar hands), and With Familiar (a stranger's hand previously familiarized in an earlier block). Participants responded via keyboard.
	(Shibuya et al., 2018)	18 healthy participants	32 electrodes (10–20 system)	Clearly stated; time- frequency analysis	Visuoproprioceptive, Visuotactile, and Visuomotor for breaking embodiment	Proprioceptive drift, and 9 questions related to SoO and SoA (7-point Likert scale)	Within-subject design; RHI of the left hand using a monitor. Participants sat with their gloved left hand hidden beneath a shelf-mounted monitor displaying a life-sized, top-down video of a gloved model hand. In a randomly ordered, synchronous and asynchronous conditions were administered. At the end of asynchronous trials, the model's hand performed an unexpected finger movement.
No-VR settings	(Blefari et al., 2011)	5 healthy, right-handed participants	16 electrodes (10–20 system)	Clearly stated; frequency analysis	Visuoproprioceptive and Visuotactile	9 questions related to SoO (7-point Likert scale)	Within-subject design; RHI of the left hand. Participants underwent a control condition, where asynchronous brush strokes were applied, and an experimental condition, with synchronous brush strokes. Each condition was repeated three times, in a fixed (non-randomized) order, with a 15-second resting period before each trial.
	(Sakamoto and Ifuku, 2021)	30 healthy, naive male participants	C4 and Fpz electrodes (10–20 system)	Not stated; time analysis	Visuoproprioceptive and Visuotactile	8 questions related to SoO (10-point Likert scale)	Within-subject design; RHI of the left hand. Participants had their real left hand hidden and a rubber hand placed in a spatially congruent position. In Experiment 1, electrical stimulation was applied to the left median nerve across four randomized conditions: (1) congruent stroking of real and rubber hands, (2) incongruent stroking, (3) viewing a cube in place of the rubber hand while receiving tactile stimulation, and (4) viewing the cube with no stimulation. In Experiment 2, only congruent and rest conditions were used, and participants pressed a button with their right hand whenever they felt ownership over the rubber hand.
	(Hiramoto et al., 2017)	26 healthy participants	64 electrodes (10–10 system)	Clearly stated; time- frequency analysis	Visuoproprioceptive and Visuotactile	5 questions related to SoO (visual analog scale: 0–100), without distinguishing between conditions.	Within-subject design; RHI of the left hand. Small vibrators were placed on the index and ring fingers of the hidden left hand, while LEDs were displayed on the corresponding fingers of the visible rubber hand. Participants were randomly presented with spatially congruent or incongruent visuo-tactile stimulation and were asked to indicate which finger they felt the vibration in by pressing a key with their right hand, while listening to white noise.
	(Shimizu et al., 2021)	9 healthy participants	8 electrodes (10–20 system)	Not stated; frequency analysis	Visuomotor	2 questions related to SoA and SoO (7-point Likert scale)	Within-subject design; Computer screen with cursor. Participants performed a dust disposal task, using a mouse to control a cursor to drag and drop virtual dust into a trash icon at the center of the screen. The cursor's movement direction was systematically altered relative to the participant's input, with five angular deviations (0°, 5°, 15°, 30°, and 45°) introduced to create varying levels of visuomotor mismatch.
	(Hsu et al., 2022)	24 healthy, right-handed participants	64 electrodes (10–20 system)	Clearly stated; time- frequency analysis (cross -frequency coupling)	Visuoproprioceptive and Visuotactile	Pressing a pedal when feeling the illusion, and a questionnaire (10-point Likert scale)	Between-subject design; traditional RHI (left hand). The experiment had two stages. In Stage 1, participants experienced synchronous and asynchronous brush strokes during the traditional RHI to assess their susceptibility to the illusion. In Stage 2, only synchronous stroking was used while EEG was recorded across five trials to compare intrinsic neural activity between high and low susceptibility groups.
	(Shibuya and Ohki, 2023)	18 healthy participants	16 electrodes (10–20 system)	Clearly stated; time- frequency analysis	Visuoproprioceptive, Visuotactile and Visuomotor	8 questions related to SoO and SoA (7-point Likert scale)	Within-subject design; RHI of the left hand using a mirror illusion. Participants viewed a mirrored rubber hand as their own, with their real left hand hidden. Four randomized conditions (2 × 2 design) varied body continuity (forearm present/absent) and stimulation (synchronous brushing or none). Each condition was randomly presented and included three phases: forearm observation, hand observation (with or without brushing), and movement observation (rubber hand wrist rotation).
	(Sciortino and Kayser, 2022a)	24 healthy, right-handed participants	128 electrodes (radial layout)	Clearly stated; time multivariate analysis	Visuopropioceptive and Visuotactile	Skin conductance, 9 questions related to SoO (7-point Likert scale), and press a computer key when feeling embodied	Within-subject design; RHI of the left hand. Participants sat at a table and were exposed to five conditions in a pseudo-randomized order: Illusion Hand Next (rubber hand placed next to the real hand in an anatomically congruent position); Incongruent Hand Next (rubber hand placed next to the real hand but rotated 90°); Illusion Hand Under (rubber hand placed directly above the real hand in a congruent orientation); Incongruent Hand Under (rubber hand placed above the real hand but rotated 90°); and Real Condition (no rubber hand). In all conditions, visuotactile stimulation was applied synchronously using small vibrators and LEDs on the index finger.
	(Rao and Kayser, 2017)	32 healthy, right-handed participants	64 electrodes (10-20 system)	Clearly stated; time and time- frequency analyses	Visuopropioceptive and Visuotactile	Press a key when feeling the illusion, and another key when the feeling is lost	Within-subject design; RHI of the left hand. Participants underwent five randomized conditions: classic RHI setup (Illusion), a rotated hand (Incongruent), no rubber hand (Real), hidden real hand below the rubber one (Hand Under), and stimulation on the contralateral hand (Two Hands). Stimuli were delivered via synchronized LED and vibration motors. The experiment was split into two parts: Experiment 1 included one repetition per condition; Experiment 2 included 3 repetitions of each condition.
	(Faivre et al., 2017)	23 healthy, right-handed participants	64 electrodes (10-20 system)	Clearly stated; frequency, source and connectivity analyses	Visuotactile	Proprioceptive drift, and 1 question related to tactile sensation (1–10 scale)	Within-subject design; self-touch RHI (sRHI) of the right hand. Participants, blindfolded and in a dark room, stroked a right rubber hand while the experimenter simultaneously stroked their own crossed right hand. There were two conditions: synchronous and asynchronous strokes. Before the stroking phase, a flash induced a negative afterimage on the participant's real hand. After 15s of stroking, participants opened their eyes and reported the perceived location of the afterimage and how strongly they felt they were touching their own hand.
	(Veillette et al., 2023)	23 participants	64 electrodes (10–20 system)	Clearly stated; time- resolved decoding and fractal analysis	Motor-based temporal congruency and prediction cues	1 question related to SoA (Yes or No answer)	Within-subject design. Participants sat at a table with a button under their right hand and a screen in front of them. There were three blocks: Blocks 1 and 3 (Baseline, 30 trials each): A fixation cross appeared, followed by a random delay, then a “Go” cue. Participants pressed the button voluntarily, and the trial ended with another fixation cross. Block 2 (EMS Block, 300 trials): After the “Go” cue, electrical muscle stimulation was applied to involuntarily move the participant's finger to press the button. EMS timing was adjusted trial-by-trial using Bayesian optimization to find where participants perceived 50% of actions as self-caused.
	(Della Longa et al., 2021)	40 children (21 preterm and 19 full-term)	128 electrodes (approx. 10–10 system)	Clearly stated; frequency analysis	Visuotactile	Proprioceptive drift, and 2 questions about SoO (7-point Likert scale)	Within-subject design; RHI of the left hand. Participants were children aged 6–11 years, including both preterm and full-term groups. Each participant underwent the RHI under two conditions: synchronous and asynchronous visuotactile stimulation.
	(Sebastiano et al., 2024)	18 (somotosensorial cues) +17 (visual cues) healthy, right-handed participants	32 electrodes (10-20 system)	Clearly stated; time analysis	Visuotactile and somatosensorial	3 questions related to embodiment and 3 questions related to desembodiment (7-point Likert scale)	Within-subject design; RHI of the right hand. Participants underwent three randomized sessions (synchronous RHI, asynchronous RHI, and baseline), each with 100 trials split into three blocks: (1) 12s of RHI induction (or rest for baseline), (2) 10 somatosensory (median nerve stimulation, Experiment 1) or visual (LED flash, Emperiment 2) stimuli at 1.5 Hz, and (3) again 12s of RHI induction. Embodiment ratings were collected after each RHI condition.
	(Sciortino and Kayser, 2022b)	24 participants	128 electrodes (radial layout)	Clearly stated; time- frequency analysis	Visuopropioceptive and Visuotactile	Press a computer key when feeling the illusion, and 9 questions related to SoO (7-point Likert scale)	Within-subject design; RHI of the left hand. Participants underwent each of the following five conditions four times in a pseudo-random order: Illusion Hand Next (rubber hand placed next to the participant's hidden real hand in a congruent position), Illusion Hand Under (rubber hand placed above the participant's real hand in a congruent position), Incongruent Hand Next (same as Illusion Hand Next, but the rubber and was rotated 90°), Incongruent Hand Under (same as Illusion Hand Under, but the rubber hand was rotated 90°), Real Hand (no rubber hand). All conditions involved synchronous visuo-tactile stimulation using a vibration motor and LED flashes.
	(Heldmann et al., 2024)	23 healthy, right-handed participants	32 electrodes (10–20 system)	Clearly stated; time and location analyses	Visuotactile	3 questions related to SoO (7-point Likert scale)	Within-subject design; RHI of the right hand. Participants completed 40 randomly presented trials (20 synchronous and 20 asynchronous). Each trial consisted of 55 brush strokes, followed by 5 test trials in which a needle was pressed on the rubber hand, and concluded with an embodiment questionnaire.
AR settings	(Hansford et al., 2023)	48 healthy participants	64 electrodes (10–20 system)	Clearly stated; time- frequency analysis)	Visuoproprioceptive and Visuotactile	6 questions related to SoO, desownership, and control (7-point Likert scale)	Participants were seated with their right hand placed inside an AR system, observing their hand's reflection through the system. Seven conditions were presented in random order: (1–2) participants saw and felt their fingers being pushed or pulled; (3–4) participants only saw the push or pull without feeling it; (5–6) participants felt the push or pull, but the visual feedback was incongruent; (7) control condition with no visual or tactile stimulation.

Across environments, 15 studies employed immersive VR, 5 used non-immersive VR, 14 relied on non-VR settings, and 1 study was conducted in AR. Regarding participants, most studies included healthy, naïve, right-handed individuals with balanced gender distributions. Only 1 study tested exclusively females, 1 exclusively males, and another focused on children, comparing full-term and pre-term participants. Group sizes ranged from 5 to 48 subjects, with an average of 24 ± 9 participants.

**Figure 3:**
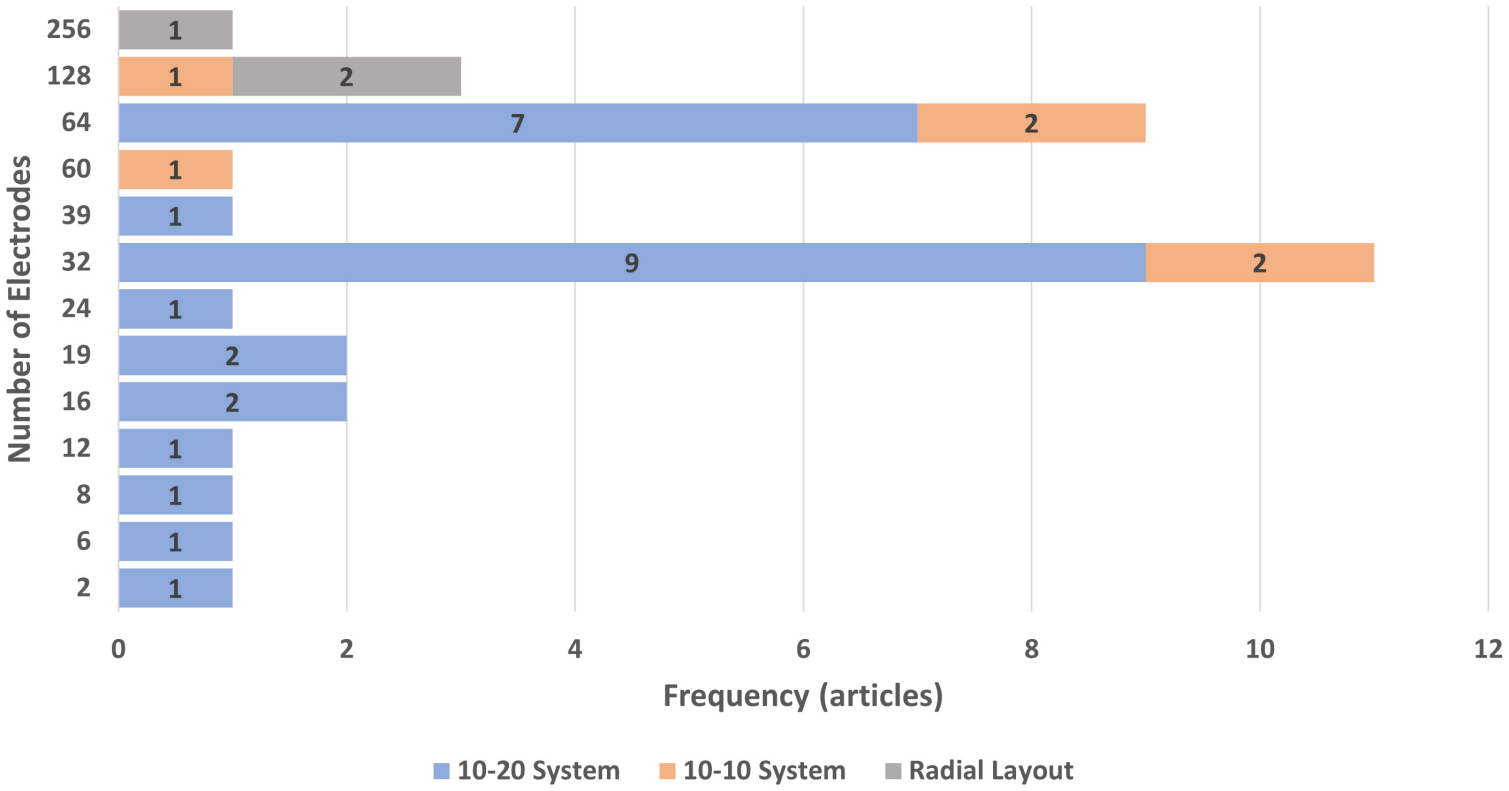
Bar chart showing the number of electrodes used in EEG recordings during embodiment illusion experiments across the included studies. Blue bars represent studies using the 10–20 placement system, orange bars represent those using the 10–10 system, and gray bars represent those using a radial layout.

EEG configurations also varied considerably (Figure 3). The majority of studies applied the 10–20 electrode placement system (11 in immersive VR, 4 in non-immersive VR, 10 in non-VR, and 1 in AR). Others used 10–10 systems or radial layouts for higher-density recordings. While 32- and 64-channel setups were most common (11 and 9 studies, respectively), channel counts ranged from as few as 6 to as many as 256. The single most frequent configuration was a 32-channel, 10–20 montage, reported in 9 studies. Importantly, this variability in electrode density and configuration did not appear to systematically depend on the environment (immersive VR, non-immersive VR, non-VR, or AR). Furthermore, only three studies (Gonzälez-Franco et al., 2014; Sakamoto and Ifuku, 2021; Shimizu et al., 2021) did not clearly report the EEG preprocessing methods applied, while the remaining 32 implemented clean and methodologically valid pipelines. Most preprocessing approaches included filtering, artifact rejection (using multiple strategies such as EOG, EMG, ICA, and visual inspection), and epoching with baseline correction. Additionally, EEG analyses included time-domain, frequency-domain, and time-frequency methods. While there was some variability in the specific EEG features analyzed (Table 2) and processing pipelines applied, this reflects the exploratory nature of the field and does not undermine the overall methodological rigor of the studies (Section 3.2.1).

All studies assessed the SoE using questionnaires, most based on Likert scales. Yet, the exact items and number of questions varied, meaning each study relied on a slightly different self-report tool. Moreover, various studies have only focused on one or more different SoE components (SoA or SoO), without looking for the full extent of the illusion as it is defined. In addition, six studies complemented questionnaires with objective measures, namely, proprioceptive drift (1 immersive VR, 3 non-immersive VR, and 2 non-VR) and one with skin conductance (non-VR).

**Figure 4:**
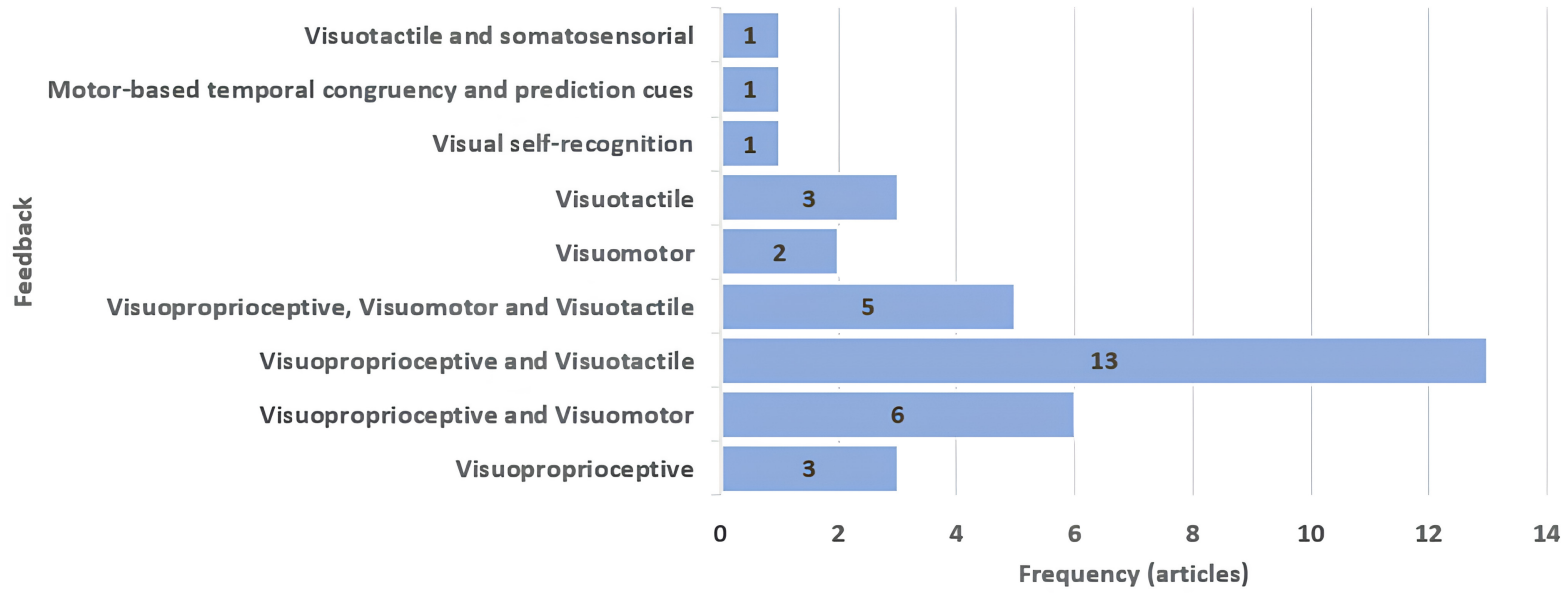
Bar chart showing the types and combinations of multisensory feedback used to induce the SoE illusion in the included studies.

Regarding induction triggers (Figure 4), most studies combined visuoproprioceptive cues with visuotactile or visuomotor feedback. However, only 5 studies (3 immersive VR, 1 non-immersive VR, and 1 non-VR) employed all three modalities simultaneously. Three studies relied solely on visuoproprioceptive cues, while 8 did not rely on visuoproprioceptive triggers at all. Thus, the type and combination of cues varied substantially across studies.

For the immersive VR settings, most studies simulated realistic virtual environments resembling physical reality, and only one study applied a between-subjects design, while all others controlled for inter-subject variability. Regarding the non-immersive studies, one used a large projection screen, while others used standard computer monitors, with two of those based on adaptation of the traditional RHI, and all applying within-subjects designs. Non-VR studies also largely applied within-subjects designs (13 of 14), with 10 adapting the left-hand RHI, 2 the right-hand RHI, and 2 designing individualized tasks. Lastly, the AR study adapted the right-hand RHI in a within-subjects design. The majority of the articles employed randomized conditions.

Overall, the majority of studies implemented some adaptation of the RHI, though with meaningful differences in procedure and cues provided for the SoE illusion. Control conditions also lacked standardization. Some studies used the absence of sensory cues, while others explicitly disrupted the illusion by delivering incongruent cues. Although often treated as equivalent, these two approaches may yield distinct neural responses. The lack of a consistent definition and implementation of control conditions highlights the urgent need for clearer methodological guidelines in SoE research.

#### 3.2.1 Quality assessment across studies

The results of the JBI critical appraisal checklist are provided in the Supplementary material. In general, the main source of potential bias across studies stems from the lack of clearly defined participant inclusion and exclusion criteria. Most studies reported basic characteristics such as health status or handedness. Although this may be acceptable for exploratory research, it does not provide information on relevant factors such as neurological history, which could influence outcomes. Additionally, some studies (Miremadi et al., 2025; Galigani et al., 2021) lacked clear methods to directly assess SoE (Table 3), whereas others (Veillette et al., 2023; Sciortino and Kayser, 2022b) provided only minimal description of the participant population.

Notable, 4 of the included studies were judged to have a moderate-to-high risk of bias. Gonzälez-Franco et al. (2014) lacked rigor in participant selection, analyzed only right-hand tasks (dominant hand) without addressing potential lateralization confounds, and did not report EEG preprocessing or analysis (referring instead to prior studies). Ramírez-Campos et al. (2024) relied on a public dataset without detailing inclusion/exclusion criteria, explicitly assessing SoE, nor addressing confounding factors. Blefari et al. (2011) did not explicitly report inclusion/exclusion criteria, did not randomize conditions, failed to remove 50 Hz line noise, and used a broad 1–60 Hz frequency range to calculate PSD; statistics were performed at the subject level with a very small sample size (*n* = 5), and few confounders were identified and not addressed. Finally, Shimizu et al. (2021) did not clearly report inclusion criteria or sample characteristics, only partially controlled for task order and session effects, did not fully identify EEG-specific confounders, and failed to describe EEG processing and statistical analysis in detail. This lack of information and no acknowledgment of confounding factors may have contribute to reporting bias, as results could selectively highlight significant findings. In contrast, six studies (Alchalabi et al., 2019; Raz et al., 2020; Esteves et al., 2025; Kang et al., 2015; Heldmann et al., 2024; Hansford et al., 2023) met all JBI criteria without any concerns flagged.

Overall, most studies presented a low to moderate risk of bias, indicating that, despite some methodological limitations, their results can be interpreted with reasonable confidence. A narrative exploration of differences across studies highlights potential sources of heterogeneity, which are expected given the exploratory nature of the included research.

## 4 Discussion

This review identifies 35 articles that focus on possible EEG correlates of the SoE over the last fifteen years, potentially indicating an objective biomarker of the illusion. The number of such studies has been increasing (Figure 2), reflecting growing interest in SoE and, consequently, in understanding how it affects brain activity. Although the number of studies is not extensive, it was possible to identify some promising features and gain an initial sense of EEG dynamics related to embodiment illusions, with several studies reporting changes in neural features, including oscillatory processes such as PSD, frequency band changes and connectivity, various evoked potentials like SEP, ERPs, and ErrPs, and signal complexity features (e.g., fractal dimension), associated with embodiment. However, this review shows that no definitive EEG biomarker of SoE has yet been established due to inconsistent findings and, in many cases, supported by only a single study (Table 2). This variability makes the current evidence base volatile and prevents strong conclusions.

Among oscillatory features, Alpha activity stands out as the most promising EEG change during SoE. Ten studies consistently reported stronger Alpha suppression over centro-parietal areas during embodiment illusions, suggesting a robust association between reduced Alpha power and SoE (Raz et al., 2020; Nicolardi et al., 2025; Kang et al., 2015; Shibuya et al., 2021, 2018; Sciortino and Kayser, 2022b; Faivre et al., 2017; Rao and Kayser, 2017; Della Longa et al., 2021; Shibuya and Ohki, 2023). Importantly, this effect was observed across diverse experimental setups (two studies using immersive VR, three using non-immersive VR, and five using non-VR paradigms), supporting the generalizability of these findings beyond a single induction method (Table 3). Alpha suppression in this context is thought to reflect increased activation of somatosensory and associative cortical areas, indicating active integration and interpretation of multisensory feedback as part of the body (Foxe and Snyder, 2011; Della Longa et al., 2021; Sciortino and Kayser, 2022b). This suggests that Alpha suppression may signal the neurophysiological processes underpinning SoE illusion formation, offering valuable insights into the cortical mechanisms of embodiment.

It is also important to consider the relationship between Alpha power and the tasks performed. Several of the tasks employed across studies involve processes such as error monitoring (Raz et al., 2020; Shibuya et al., 2021, 2018), motion or body observation (Nicolardi et al., 2025; Shibuya and Ohki, 2023), and attention or task engagement (Kang et al., 2015; Della Longa et al., 2021; Faivre et al., 2017), all of which are known to modulate Alpha activity. Consequently, the observed changes in this band may be associated, at least in part, with general immersion or task engagement. While most studies contrasted SoE and no-SoE conditions within the same task, reporting more pronounced Alpha reductions when the illusion was present compared to control conditions and therefore associating this change with SoE, these effects were consistently observed within specific task frameworks. Thus, Alpha modulation may serve as an indicator of the illusion only within specific contexts. This further underscores the importance of paradigm selection in SoE research. Nonetheless, other included studies directly associated reductions in Alpha power with embodiment illusions (Sciortino and Kayser, 2022b; Rao and Kayser, 2017), differentiating these changes from task-related effects and strengthening the reported relationship between general Alpha reduction and embodiment illusions.

At the same time, the reliability of this result is tempered by contradictory findings. The studies from Evans and Blanke (2013) and Lenggenhager et al. (2011) reported increased Alpha power, particularly in the mPFC medial prefrontal cortex and fronto-parietal regions. However, these changes were associated with specific SoE components, specifically SoSL and SoO, which may explain the discrepancies. It is plausible that different SoE components elicit distinct neural responses (Guy et al., 2023), complicating interpretation. In addition, three studies reported no significant correlation between alpha activity and SoE (Li et al., 2023; Esteves et al., 2025; Hansford et al., 2023); however, this may be result of small sample sizes, individual variability in illusion susceptibility [since personality and social traits influence SoE illusion (Dewez et al., 2019; Guy et al., 2023)], or methodological differences in the control conditions used. Since these studies used some type of VR (immersive VR and AR), this may also be an effect of the way feedback was provided to induce the illusion. Despite these inconsistencies, a total of 18 studies identified some association between Alpha rhythms and SoE (Table 2), with only one classified as having a moderate to high risk of bias (Ramírez-Campos et al., 2024; Table 4). This highlights Alpha modulation as one of the most consistent EEG signatures of embodiment illusions.

**Table 4:** Result of JBI critical appraisal checklist for analytical cross-sectional studies.

**Paradigm**	**Study**	**Q1**	**Q2**	**Q3**	**Q4**	**Q5**	**Q6**	**Q7**	**Q8**	**Comments**
Immersive VR settings	(Gonzälez-Franco et al., 2014)	Unclear	Yes	Yes	Yes	No	No	No	Yes	Participant inclusion/exclusion criteria were not explicitly reported; only the dominant hand was analyzed (lateralization not addressed); EEG preprocessing not clearly described; no non-embodiment control condition; no pain ratings; statistical methods poorly reported.
	(Jeunet et al., 2018)	Unclear	Yes	Yes	Yes	Yes	Yes	Yes	Yes	Participant inclusion/exclusion criteria were not explicitly reported; SoA was assessed using non–gold-standard methods; statistical methods poorly reported.
	(Aspell et al., 2012)	Unclear	Yes	Yes	Yes	Yes	Yes	Yes	Yes	Participant inclusion/exclusion criteria were not explicitly reported; SoE inferred from self-identification.
	(Alchalabi et al., 2019)	Yes	Yes	Yes	Yes	Yes	Yes	Yes	Yes	—
	(Porssut et al., 2023)	Unclear	Yes	Yes	Yes	Yes	Yes	Yes	Yes	Participant inclusion/exclusion criteria were not explicitly reported; SoE was assessed using non-gold-standard methods, but the choice was justified.
	(Raz et al., 2020)	Yes	Yes	Yes	Yes	Yes	Yes	Yes	Yes	—
	(Pavone et al., 2016)	Unclear	Yes	Yes	Yes	Yes	Yes	Yes	Yes	Participant inclusion/exclusion criteria were not explicitly reported; SoE inferred from avatar exposure; statistical methods poorly reported.
	(Li et al., 2023)	Unclear	Yes	Yes	Yes	Yes	Yes	Yes	Yes	Participant inclusion/exclusion criteria were not explicitly reported; control condition insufficiently described; SoE was assessed using non-gold-standard methods; unclear description of study design.
	(Evans and Blanke, 2013)	Unclear	Yes	Yes	Yes	Yes	Yes	Yes	Yes	Participant inclusion/exclusion criteria were not explicitly reported.
	(Iwane et al., 2024)	Unclear	Yes	Yes	Yes	Yes	Yes	Yes	Yes	Participant inclusion/exclusion criteria were not explicitly reported; SoE was assessed using non-gold-standard methods.
	(Ramírez-Campos et al., 2024)	Yes	Yes	Unclear	Unclear	No	No	Yes	Yes	Public dataset used; participants inclusion/exclusion criteria insufficiently reported; no explicit assessment of SoE; confounding factors not addressed.
	(Esteves et al., 2025)	Yes	Yes	Yes	Yes	Yes	Yes	Yes	Yes	—
	(Nicolardi et al., 2025)	Unclear	Yes	Yes	Yes	Yes	Yes	Yes	Yes	Participant inclusion/exclusion criteria were not explicitly reported.
	(Lu et al., 2025)	Unclear	Yes	Yes	Yes	Yes	Yes	Yes	Yes	Participant inclusion/exclusion criteria were not explicitly reported; SoE was assessed using non-gold-standard methods; some confounders not addressed.
	(Miremadi et al., 2025)	Yes	Yes	Yes	No	Yes	Yes	Yes	Yes	SoE inferred from avatar exposure; some identified confounders not addressed.
No immersive VR settings	(Lenggenhager et al., 2011)	Unclear	Yes	Yes	Yes	Yes	Yes	Yes	Yes	SoE was assessed using non-gold-standard methods.
	(Kang et al., 2015)	Yes	Yes	Yes	Yes	Yes	Yes	Yes	Yes	SoE was assessed using non-gold-standard methods.
	(Shibuya et al., 2021)	Unclear	Yes	Yes	Yes	Yes	Yes	Yes	Yes	Participant inclusion/exclusion criteria were not explicitly reported.
	(Galigani et al., 2021)	Unclear	Yes	Yes	No	Yes	Yes	Yes	Yes	Participant inclusion/exclusion criteria were not explicitly reported; SoE inferred from self-identification.
	(Shibuya et al., 2018)	Unclear	Yes	Yes	Yes	Yes	Yes	Yes	Yes	Participant inclusion/exclusion criteria were not explicitly reported; statistical methods poorly reported.
No-VR settings	(Blefari et al., 2011)	Unclear	Yes	Yes	Yes	No	No	No	Yes	Participant inclusion/exclusion criteria were not explicitly reported; statistical methods poorly reported; 50-Hz line noise was not removed; poor identification of confounding factors (only suggestibility/placebo acknowledged).
	(Sakamoto and Ifuku, 2021)	Unclear	Yes	Yes	Yes	Yes	Yes	Unclear	Yes	Participant inclusion/exclusion criteria were not explicitly reported; EEG acquisition and SEP measurement based on established methods, but not detailed; key confounds (attention, handedness, prior RHI experience) not addressed.
	(Hiramoto et al., 2017)	Unclear	Yes	Yes	Yes	Yes	Yes	Yes	Yes	Participant inclusion/exclusion criteria were not explicitly reported; conditions could not be distinguished by subjective reports, resulting in a single rating for both, but this limitation was acknowledged.
	(Shimizu et al., 2021)	No	No	Yes	Yes	Yes	No	No	No	Participant inclusion/exclusion criteria were not explicitly reported; sample characteristics minimal; EEG confounders not fully addressed; preprocessing/statistical adjustments unclear; ANOVA and discriminant analysis appropriate but limited by small sample size and lack of assumption checks or multiple comparison correction.
	(Hsu et al., 2022)	Unclear	Yes	Yes	Yes	Yes	Yes	Yes	Yes	Participant inclusion/exclusion criteria were not explicitly reported; population poorly described; between-subjects design partially controlled via preliminary stage.
	(Shibuya and Ohki, 2023)	Unclear	Yes	Yes	Yes	Yes	Yes	Yes	Yes	Participant inclusion/exclusion criteria were not explicitly reported; attention, visual alpha contamination, and kinesthetic expectations were not controlled as confounding factors, although some were acknowledged.
	(Sciortino and Kayser, 2022a)	Unclear	Yes	Yes	Yes	Yes	Yes	Yes	Yes	Preliminary testing ensured RHI perception, but participant inclusion/exclusion criteria were not explicitly reported; sample characteristics minimal.
	(Rao and Kayser, 2017)	Unclear	Yes	Yes	Yes	Yes	Yes	Yes	Yes	Preliminary testing ensured RHI perception, but participant inclusion/exclusion criteria were not explicitly reported; SoE was assessed using non-gold-standard methods.
	(Faivre et al., 2017)	Unclear	Yes	Yes	Yes	Yes	Yes	Yes	Yes	Participant inclusion/exclusion criteria were not explicitly reported; SoE was assessed using non-gold-standard methods.
	(Veillette et al., 2023)	Unclear	No	Yes	Yes	Yes	Yes	Yes	Yes	Participant inclusion/exclusion criteria were not explicitly reported; sample not reported; SoA was assessed using non-gold-standard methods; block order not randomized (baseline-condition-baseline).
	(Della Longa et al., 2021)	Unclear	Yes	Yes	Yes	Yes	Yes	Yes	Yes	Participant inclusion/exclusion criteria were not explicitly reported; EEG confounds in children (e.g., head movement) not addressed.
	(Sebastiano et al., 2024)	Unclear	Yes	Yes	Yes	Yes	Yes	Yes	Yes	Participant inclusion/exclusion criteria were not explicitly reported.
	(Sciortino and Kayser, 2022b)	Yes	No	Yes	Yes	Yes	Yes	Yes	Yes	Participant inclusion/exclusion criteria were not explicitly reported.
	(Heldmann et al., 2024)	Yes	Yes	Yes	Yes	Yes	Yes	Yes	Yes	Analysis time frame chosen by visual inspection.
AR settings	(Hansford et al., 2023)	Yes	Yes	Yes	Yes	Yes	Yes	Yes	Yes	Acknowledged potential confounds (IC, illusion susceptibility, EEG variability) without correction.

Beta and gamma power have also been investigated, with several studies reporting decreased beta and increased gamma activity during the illusion. However, these results were contradicted by a comparable number of studies showing no differences, or even opposite effects, reducing confidence in their reliability. These frequency bands are nonetheless theoretically relevant, as they subserve distinct neurophysiological functions that could support the mechanisms of SoE illusions. Beta activity has been linked to multisensory conflict resolution, sensory integration, and network coordination for predicting perceptual outcomes (Esteves et al., 2025; Romero et al., 2015; Hipp et al., 2011). All of these processes are critical for constructing the illusion, reinforcing the potential involvement of beta oscillations in embodiment. Gamma activity, in turn, plays a role in multisensory integration and cross-modal binding (Esteves et al., 2025; Kanayama et al., 2007), as well as in connectivity between sensorimotor and parietal cortices during hand ownership (Faivre et al., 2017). This suggests that Gamma oscillations may facilitate inter-regional information transfer necessary for sustaining the illusion. Thus, even if the precise influence of Beta and Gamma activity on SoE remains insufficiently defined, the positive findings reported point to important roles of these bands in embodiment illusions, underscoring their potential as EEG biomarkers worthy of further investigation.

Additionally, several studies have underscored the temporal evolution of SoE illusions, suggesting that this dynamic process can influence EEG activity, as different time points during the illusion may elicit distinct neural responses. Kilteni et al. (2012b) proposed that the relative importance of the three components of embodiment (SoO, SoA, and SoSL) may vary over time or depend on the specific experimental context. Together with evidence showing that these components are associated with distinct brain regions (Guy et al., 2023), this supports the possibility that different moments within the illusion may be reflected in the EEG signal in different ways. As argued by Esteves et al. (2025), SoE appears to be a dynamic, fast-evolving process that emerges within seconds and strengthens over time. This suggests embodiment is underpinned by distributed and transient neural interactions, rather than a stable single-band predictor, complicating the identification of a definitive EEG biomarker. Therefore, contradictory or the absence of clear results in some studies may also be influenced by the temporal evolution of SoE, a factor that has been largely overlooked in the existing literature.

Regarding Delta and Theta power, only a few studies have addressed these bands, with most reporting no effect or contradictory observations. This creates a gap in the research field, preventing firm conclusions about their relationship with SoE. Nonetheless, some evidence suggests possible links between SoE and Delta/Theta modulation. For example, Takeo et al. (2025) used immersive VR to investigate the neural mechanisms of thermal perception during embodiment and identified Theta-Band activity in the insula as a correlate of multisensory body-related processing, essential to the illusion. Similarly, Esteves et al. (2025) reported decreases in occipital Delta power during embodiment, although these effects did not reach statistical significance. In addition, Hsu et al. (2022) showed that cross-frequency communication between Delta and Alpha bands influences susceptibility to the illusion, with Delta modulating Alpha power over frontal regions. Finally, Ramírez-Campos et al. (2024) also reported associations between SoE and theta-band activity over central regions, further suggesting a potential role for low-frequency oscillations. However, this study was judged to have a moderate to high risk of bias, which limits the strength of the conclusions that can be drawn from these findings. Thus, the current evidence remains sparse and methodologically heterogeneous, and conclusions are constrained by the limited number of studies and, in some cases, elevated risk of bias. Taken together, these findings highlight the potential relevance of lower-frequency oscillations for embodiment illusions, reinforcing that delta and theta activity may play a meaningful role in SoE and should not be overlooked in future research. Future research employing standardized paradigms, rigorous EEG methodologies, and clearly defined embodiment assessments is needed to clarify the role of low-frequency oscillations in SoE.

Other EEG features, such as SEP, ErrPs, other ERPs, and fractal dimension, also show possible changes associated with SoE. However, since each of these features was investigated by only one study, the evidence is too limited to draw conclusions.

Overall, these findings suggest that while certain EEG features, particularly Alpha suppression, hold potential, the current literature does not support a robust, universal biomarker of embodiment. Accordingly, these findings should be considered in light of the methodological quality of the included studies. Although most studies were judged to have a low to moderate risk of bias (Table 4), several presented limitations. In particular, the majority of studies did not clearly report participant inclusion and exclusion criteria, limiting the characterization of the studied populations and potentially introducing uncontrolled variability. In addition, some studies showed limitations in the clarity or validity of SoE assessment, which may reflect the fact that embodiment was not always the primary research focus, while others studies also failed to identify or adequately address relevant confounding factors, making it necessary to interpret findings within the specific experimental paradigms employed. Crucially, the most convergent finding (Alpha suppression over centro-parietal regions) were predominantly reported by studies with low risk of bias, strengthening confidence in this association. In the end, the risk-of-bias assessment reveals that several EEG signatures of SoE appear robust across methodologically sound studies, while other reported effects remain preliminary and require confirmation in better-controlled and more standardized experimental designs. Additionally, the diverse paradigms and data collection methods applied likely introduces further sources of bias, complicating cross-study comparisons and contributing to inconsistency.

First, most studies recruited small, homogeneous samples of young, healthy university students, limiting generalizability and reducing statistical power (Table 3). Second, task designs and induction methods varied widely across immersive VR, non-immersive VR, AR, and non-VR paradigms. While many relied on RHI adaptations, the types of cues (visuoproprioceptive, visuotactile, visuomotor, etc.) differed (Figure 4). Even control conditions were also inconsistent: some studies introduced feedback specifically designed to disrupt SoE, while others provided no feedback at all. Although both approaches aim to prevent SoE induction, these inconsistencies may strongly influence neural responses, since SoE depends heavily on the type of feedback provided (Falcone et al., 2023). Disruptive feedback may engage additional neural processes related to sensory conflict, prediction error, or error monitoring, whereas no feedback conditions may instead reflect a passive baseline in which embodiment simply do to emerge. As a result, EEG differences between experimental and control conditions could reflect not only the presence or absence of SoE, but also neural activity associated with feedback conflict or sensory disruption. As argued in the literature, the way feedback is delivered can modulate neural activity differently, and EEG changes should be interpreted in light of this feedback processing. Furthermore, since SoE is a perception illusion, the multisensory cues provided, even during control conditions, can change how subjects see and feel the illusion, further difficult the standardization of SoE easement through questionnaires. Third, outcome measures of SoE were highly heterogeneous (Table 3). Although all studies used self-report questionnaires, there was substantial variation in items and scales. Only a minority included objective measures, such as proprioceptive drift (6 studies) or physiological indices like skin conductance (1 study). This lack of standardization in the evaluation of SoE further contributes to divergent findings. The inherent subjectivity of the questionnaires creates its own limitations, but by employing different questionnaires, this is further exacerbated. Lastly, EEG methodologies also lacked uniformity, with electrode setups ranging from sparse (6 channels) to high-density (256 channels) arrays (Figure 3). The EEG processing steps employed, although methodologically valid, varied considerably across studies, largely due to differences in the EEG features investigated and the underlying hypotheses. This heterogeneity further complicates cross-study comparisons and limits the strength of the conclusions that can be drawn. PSD, ERP, SEP, and ErrP analyses were all applied, but few studies examined features in directly comparable ways. Although 35 studies investigated EEG changes during SoE, only a handful reported overlapping results. Moreover, it's also important to consider that non-significant findings were often underreported, increasing the risk of selective reporting bias.

Taken together, these issues introduce a risk of bias in the current literature. With the growing adoption of VR and the increasing relevance of embodiment in health fields such as neurorehabilitation and psychological therapies, SoE research is likely to expand, creating the need to understand how SoE influences brain dynamics, to define an objective SoE biomarker, and to standardize guidelines for inducing and evaluating SoE illusions. However, the current literature remains limited by homogeneous samples, methodological heterogeneity, and a lack of standardized assessment tools. To strengthen reliability and comparability, future studies should prioritize larger, preregistered designs, adopt a universal questionnaire to evaluate SoE, and establish or follow standardized induction and control paradigms—for example, the seven-step toolbox for designing and assessing embodiment experiences proposed by Falcone et al. (2023). Efforts should also focus on replicating and refining previous findings to move toward a robust and reliable EEG biomarker of embodiment. Finally, the limited number of studies (only 35) underscores the need for further exploration in ecologically valid contexts. Moreover, current findings suggest that SoE may emerge from distributed and dynamic neural interactions, rather than a single frequency-band predictor, highlighting the need for multimodal approaches that combine oscillatory dynamics, ERP markers, and behavioral or physiological measures, and that also consider the temporal evolution of SoE.

In conclusion, and to directly address the research questions, no consistently reported EEG biomarker or standardized procedure for inducing and evaluating SoE-related EEG changes currently exists. Still, the most convergent evidence points to more accentuated Alpha suppression over centro-parietal sites as the strongest candidate, with the most common paradigm involving adaptations of the RHI that combine visuoproprioceptive and visuotactile cues. Typically, SoE was assessed using Likert-scale questionnaires, and EEG was recorded with 32 electrodes in a 10–20 layout.

Additionally, this conclusion should be interpreted with caution, as some methodological limitations must be acknowledged. The most notable limitation is that study screening, eligibility assessment, and data extraction were conducted by a single reviewer, which may have introduced bias due to subjective interpretation, particularly given the exploratory nature of the review. This may also have influenced the qualitative narrative synthesis of the results. Furthermore, the findings rely on qualitative convergence rather than statistical aggregation, owing to the substantial heterogeneity in experimental paradigms and reported outcomes. Then, no formal assessment of reporting bias or overall certainty of the evidence was conducted, which may affect confidence in the interpretation of the findings. Moreover, the review excluded gray literature and earlier publications (prior to 2010). Although this search window is justified in the context of the research question, embodiment illusions emerged and were first defined around 1998 with the RHI (Botvinick and Cohen, 1998); therefore, some relevant studies may fall outside the selected time frame. Despite these limitations, the review provides a well-structured overview of the existing literature, offering an initial synthesis of EEG correlates of embodiment illusions and aiming to support and guide future research in this field.

### 4.1 Best-practice recommendations

Given the observed variability in paradigms and methods used to explore SoE, direct comparisons across studies and results are often challenging. Therefore, researchers should work collaboratively toward greater standardization in EEG-based SoE research.

First, given the strong potential of VR to induce and manipulate SoE, we recommend the use of VR environments in future studies, preferably employing the most complete possible paradigm, namely the inclusion of visuomotor, visuotactile, and visuoproprioceptive cues. Although the body of research reviewed here shows that the presence of all three cues is not strictly necessary to study SoE illusions, using different combinations of cues introduces additional sources of variability, as the interaction between cues and their individual contributions to SoE are not yet fully understood (Guy et al., 2023; Segil et al., 2022). Moreover, it would also be beneficial to define a common baseline for no-embodiment conditions. We suggest ensuring that SoE does not occur toward the same virtual avatar used in the SoE induction conditions. This would reduce a source of variability and allow for larger sample sizes by enabling comparisons across control conditions from multiple studies. Regarding experimental tasks, RHI and MI paradigms were the most commonly employed and therefore represent the most standardized approaches for identifying EEG-based SoE biomarkers. Self-identification tasks may also be valuable, as they explicitly engage processes related to body representation and self-perception, which are central to SoE illusions. Concerning EEG signal analysis, the wide variety of analytical approaches and findings reported in the literature underscores the need for continued exploration of diverse hypotheses. Oscillatory processes were the most frequently investigated features across studies and appear to be the most promising candidates for SoE-related biomarkers. Their relatively frequent use also facilitates cross-study comparison and the accumulation of converging evidence. Nevertheless, this remains an insufficiently explored area, justifying the continued application of multiple analytical approaches. Finally, greater standardization in EEG data acquisition would be beneficial. Based on the most common practices identified in the literature, we recommend the use of at least 32 electrodes arranged according to the international 10–20 system. This configuration provides adequate scalp coverage and supports more detailed signal analyses, including source localization approaches.

## 5 Conclusion

This review included 35 studies investigating EEG correlates of the SoE illusion. Based on the available evidence, no single EEG feature can yet be regarded as a definitive biomarker of SoE. Although some studies report associations between SoE and various EEG features, such as frequency band power, band connectivity, ERPs, SEPs, and ErrPs, stronger Alpha suppression over centro-parietal regions during the illusion emerges as the most consistent finding. Nevertheless, contradictory results prevent firm conclusions. Moreover, no standardized procedure has been consistently adopted for inducing or assessing EEG-based SoE biomarkers. Experimental paradigms (including feedback modalities and task designs), control conditions, and outcome measures remain highly heterogeneous. For SoE assessment, subjective questionnaires are commonly used, but they vary considerably across studies, while objective measures are only sporadically applied. Given that SoE is highly sensitive to the sensory and contextual triggers provided, such variability poses a major challenge for comparing results across studies. These issues are particularly significant considering that only 35 studies to date have examined EEG biomarkers of SoE, with just 20 conducted in VR environments (across both immersive and non-immersive settings). Consequently, the field remains underexplored, especially in ecologically valid VR contexts. Further research is therefore essential to establish more robust and generalizable findings. Ultimately, developing a reliable framework for studying SoE and identifying effective EEG biomarkers of embodiment will require coordinated methodological efforts and replication across diverse experimental settings.

## Data Availability

The original contributions presented in the study are included in the article/Supplementary material, further inquiries can be directed to the corresponding author.
