# Supplementary material for: EEG biomarkers of the sense of embodiment: methodological gaps and evidence-based recommendations from a systematic review
Source: Front Syst Neurosci. 2026 Mar 20;20:1756407. doi: 10.3389/fnsys.2026.1756407 (PMC13047143; doi:10.3389/fnsys.2026.1756407)
Supplement: Supplementary file 1 [file Data_Sheet_1.pdf]

Supplementary Material

Table 1: Result of JBI critical appraisal checklist for analytical cross-sectional studies.

	Study	Q1	Q2	Q3	Q4	Q5	Q6	Q7	Q8	Comments
**Immersive VR settings**	González-Franco et al. (2014)	Unclear	Yes	Yes	Yes	No	No	No	Yes	Participant inclusion/exclusion criteria were not explicitly reported; only the dominant hand was analyzed (lateralization not addressed); EEG preprocessing not clearly described; no non-embodiment control condition; no pain ratings; statistical methods poorly reported.
	Jeunet et al. (2018)	Unclear	Yes	Yes	Yes	Yes	Yes	Yes	Yes	Participant inclusion/exclusion criteria were not explicitly reported; SoA was assessed using non–gold-standard methods; statistical methods poorly reported.
	Aspell et al. (2012)	Unclear	Yes	Yes	Yes	Yes	Yes	Yes	Yes	Participant inclusion/exclusion criteria were not explicitly reported; SoE inferred from self-identification.
	Alchalabi et al. (2019)	Yes	Yes	Yes	Yes	Yes	Yes	Yes	Yes	—
	Porssut et al. (2023)	Unclear	Yes	Yes	Yes	Yes	Yes	Yes	Yes	Participant inclusion/exclusion criteria were not explicitly reported; SoE was assessed using non–gold-standard methods, but the choice was justified.
	Raz et al. (2020)	Yes	Yes	Yes	Yes	Yes	Yes	Yes	Yes	—
	Pavone et al. (2016)	Unclear	Yes	Yes	Yes	Yes	Yes	Yes	Yes	Participant inclusion/exclusion criteria were not explicitly reported; SoE inferred from avatar exposure; statistical methods poorly reported.
	Li et al. (2023)	Unclear	Yes	Yes	Yes	Yes	Yes	Yes	Yes	Participant inclusion/exclusion criteria were not explicitly reported; control condition insufficiently described; SoE was assessed using non-gold-standard methods; unclear description of study design.
	Evans and Blanke (2013)	Unclear	Yes	Yes	Yes	Yes	Yes	Yes	Yes	Participant inclusion/exclusion criteria were not explicitly reported.
	Iwane et al. (2024)	Unclear	Yes	Yes	Yes	Yes	Yes	Yes	Yes	Participant inclusion/exclusion criteria were not explicitly reported; SoE was assessed using non-gold-standard methods.
	Ramírez-Campos et al. (2024)	Yes	Yes	Unclear	Unclear	No	No	Yes	Yes	Public dataset used; participants inclusion/exclusion criteria insufficiently reported; no explicit assessment of SoE; confounding factors not addressed.
	Esteves et al. (2025)	Yes	Yes	Yes	Yes	Yes	Yes	Yes	Yes	—
	Nicolardi et al. (2025)	Unclear	Yes	Yes	Yes	Yes	Yes	Yes	Yes	Participant inclusion/exclusion criteria were not explicitly reported.
	Lu et al. (2025)	Unclear	Yes	Yes	Yes	Yes	Yes	Yes	Yes	Participant inclusion/exclusion criteria were not explicitly reported; SoE was assessed using non-gold-standard methods; some confounders not addressed.
	Miremadi et al. (2025)	Yes	Yes	Yes	No	Yes	Yes	Yes	Yes	SoE inferred from avatar exposure; some identified confounders not addressed.
**No immersive VR settings**	Lenggenhager et al. (2011)	Unclear	Yes	Yes	Yes	Yes	Yes	Yes	Yes	SoE was assessed using non-gold-standard methods.
	Kang et al. (2015)	Yes	Yes	Yes	Yes	Yes	Yes	Yes	Yes	SoE was assessed using non-gold-standard methods.
	Kang et al. (2015)	Unclear	Yes	Yes	Yes	Yes	Yes	Yes	Yes	Participant inclusion/exclusion criteria were not explicitly reported.
	Shibuya et al. (2021)	Unclear	Yes	Yes	No	Yes	Yes	Yes	Yes	Participant inclusion/exclusion criteria were not explicitly reported; SoE inferred from self-identification.
	Shibuya et al. (2018)	Unclear	Yes	Yes	Yes	Yes	Yes	Yes	Yes	Participant inclusion/exclusion criteria were not explicitly reported; statistical methods poorly reported.

Table 2: Result of JBI critical appraisal checklist for analytical cross-sectional studies (Continuation).

	Study	Q1	Q2	Q3	Q4	Q5	Q6	Q7	Q8	Comments
**No-VR settings**	Blefari et al. (2011)	Unclear	Yes	Yes	Yes	No	No	No	Yes	Participant inclusion/exclusion criteria were not explicitly reported; statistical methods poorly reported; 50 Hz line noise was not removed; poor identification of confounding factors (only suggestibility/placebo acknowledged).
	Sakamoto and Ifuku (2021)	Unclear	Yes	Yes	Yes	Yes	Yes	Unclear	Yes	Participant inclusion/exclusion criteria were not explicitly reported; EEG acquisition and SEP measurement based on established methods, but not detailed; key confounds (attention, handedness, prior RHI experience) not addressed.
	Hiramoto et al. (2017)	Unclear	Yes	Yes	Yes	Yes	Yes	Yes	Yes	Participant inclusion/exclusion criteria were not explicitly reported; conditions could not be distinguished by subjective reports, resulting in a single rating for both, but this limitation was acknowledged.
	Shimizu et al. (2021)	No	No	Yes	Yes	Yes	No	No	No	Participant inclusion/exclusion criteria were not explicitly reported; sample characteristics minimal; EEG confounders not fully addressed; preprocessing/statistical adjustments unclear; ANOVA and discriminant analysis appropriate but limited by small sample size and lack of assumption checks or multiple comparison correction.
	Hsu et al. (2022)	Unclear	Yes	Yes	Yes	Yes	Yes	Yes	Yes	Participant inclusion/exclusion criteria were not explicitly reported; population poorly described; between-subjects design partially controlled via preliminary stage.
	Shibuya and Ohki (2023)	Unclear	Yes	Yes	Yes	Yes	Yes	Yes	Yes	Participant inclusion/exclusion criteria were not explicitly reported; attention, visual alpha contamination, and kinesthetic expectations were not controlled as confounding factors, although some were acknowledged.
	Sciortino and Kayser (2022a)	Unclear	Yes	Yes	Yes	Yes	Yes	Yes	Yes	Preliminary testing ensured RHI perception, but participant inclusion/exclusion criteria were not explicitly reported; sample characteristics minimal.
	Rao and Kayser (2017)	Unclear	Yes	Yes	Yes	Yes	Yes	Yes	Yes	Preliminary testing ensured RHI perception, but participant inclusion/exclusion criteria were not explicitly reported; SoE was assessed using non-gold-standard methods.
	Faivre et al. (2017)	Unclear	Yes	Yes	Yes	Yes	Yes	Yes	Yes	Participant inclusion/exclusion criteria were not explicitly reported; SoE was assessed using non-gold-standard methods.
	John P. Veillette et al. (2023)	Unclear	No	Yes	Yes	Yes	Yes	Yes	Yes	Participant inclusion/exclusion criteria were not explicitly reported; sample not reported; SoA was assessed using non-gold-standard methods; block order not randomized (baseline - condition - baseline).
	Della Longa et al. (2021)	Unclear	Yes	Yes	Yes	Yes	Yes	Yes	Yes	Participant inclusion/exclusion criteria were not explicitly reported; EEG confounds in children (e.g., head movement) not addressed.
	Sebastiano et al. (2024)	Unclear	Yes	Yes	Yes	Yes	Yes	Yes	Yes	Participant inclusion/exclusion criteria were not explicitly reported.
	Sciortino and Kayser (2022b)	Yes	No	Yes	Yes	Yes	Yes	Yes	Yes	Participant inclusion/exclusion criteria were not explicitly reported.
	Heldmann et al. (2024)	Yes	Yes	Yes	Yes	Yes	Yes	Yes	Yes	Analysis time frame chosen by visual inspection.
**AR settings**	Hansford et al. (2023)	Yes	Yes	Yes	Yes	Yes	Yes	Yes	Yes	Acknowledged potential confounds (IC, illusion susceptibility, EEG variability) without correction.

Table 3: PRISMA 2020 Checklist.

Section and Topic	Iter	Checklist Item	Location where item is reported
TITLE			
Title	1	Identify the report as a systematic review.	Title
ABSTRACT			
Abstract	2	See the PRISMA 2020 for Abstracts Checklist.	Abstract
INTRODUCTION			
Rationale	3	Describe the rationale for the review in the context of existing knowledge.	Introduction
Objectives	4	Provide an explicit statement of the objective(s) or question(s) the review addresses.	Introduction and Methodology
METHODS			
Eligibility Criteria	5	Specify the inclusion and exclusion criteria for the review and how studies were grouped for the syntheses	Methodology (Inclusion Criteria)
Information sources	6	Specify all databases, registers, websites, organisations, reference lists and other sources searched or consulted to identify studies. Specify the date when each source was last searched or consulted.	Methodology (Search strategy)
Search strategy	7	Present the full search strategies for all databases, registers and websites, including any filters and limits used.	Methodology (Search strategy)
Selection process	8	Specify the methods used to decide whether a study met the inclusion criteria of the review, including how many reviewers screened each record and each report retrieved, whether they worked independently, and if applicable, details of automation tools used in the process.	Methodology (Study Selection and Data Extraction)
Data collection process	9	Specify the methods used to collect data from reports, including how many reviewers collected data from each report, whether they worked independently, any processes for obtaining or confirming data from study investigators, and if applicable, details of automation tools used in the process.	Methodology (Study Selection and Data Extraction)
Data items	10a	List and define all outcomes for which data were sought. Specify whether all results that were compatible with each outcome domain in each study were sought (e.g. for all measures, time points, analyses), and if not, the methods used to decide which results to collect.	Methodology (Study Selection and Data Extraction)
	10b	List and define all other variables for which data were sought (e.g. participant and intervention characteristics, funding sources). Describe any assumptions made about any missing or unclear information.	Methodology (Study Selection and Data Extraction)
Study risk of bias assessment	11	Specify the methods used to assess risk of bias in the included studies, including details of the tool(s) used, how many reviewers assessed each study and whether they worked independently, and if applicable, details of automation tools used in the process.	Methodology (Quality Assessment)
Effect measures	12	Specify for each outcome the effect measure(s) (e.g. risk ratio, mean difference) used in the synthesis or presentation of results	Methodology (Study Selection and Data Extraction)
Synthesis methods	13a	Describe the processes used to decide which studies were eligible for each synthesis (e.g. tabulating the study intervention characteristics and comparing against the planned groups for each synthesis (item #5)).	Methodology (Study Selection and Data Extraction)
	13b	Describe any methods required to prepare the data for presentation or synthesis, such as handling of missing summary statistics, or data conversions.	Methodology (Study Selection and Data Extraction)
	13c	Describe any methods used to tabulate or visually display results of individual studies and syntheses.	Methodology and Results
	13d	Describe any methods used to synthesize results and provide a rationale for the choice(s). If meta-analysis was performed, describe the model(s), method(s) to identify the presence and extent of statistical heterogeneity, and software package(s) used.	Methodology (Study Selection and Data Extraction)
	13e	Describe any methods used to explore possible causes of heterogeneity among study results (e.g. subgroup analysis, meta-regression).	Not applicable
	13f	Describe any sensitivity analyses conducted to assess robustness of the synthesized results.	Not applicable
Reporting bias assessment	14	Describe any methods used to assess risk of bias due to missing results in a synthesis (arising from reporting biases).	Not applicable
Certainty assessment	15	Describe any methods used to assess certainty (or confidence) in the body of evidence for an outcome.	Not applicable

Table 4: PRISMA 2020 Checklist (Continuation).

Section and Topic	Iter	Checklist Item	Location where item is reported
RESULTS			
Study selection	16a	Describe the results of the search and selection process, from the number of records identified in the search to the number of studies included in the review, ideally using a flow diagram.	Results
	16b	Cite studies that might appear to meet the inclusion criteria, but which were excluded, and explain why they were excluded.	Results / Not applicable
Study Characteristics	17	Cite each included study and present its characteristics.	Results
Risk of bias in studies	18	Present assessments of risk of bias for each included study.	Results (Quality Assessment Across Studies)
Results of individual studies	19	For all outcomes, present, for each study: (a) summary statistics for each group (where appropriate) and (b) an effect estimate and its precision (e.g. confidence/credible interval), ideally using structured tables or plots.	Not applicable
Results of syntheses	20a	For each synthesis, briefly summarise the characteristics and risk of bias among contributing studies	Results (Quality Assessment Across Studies)
	20b	Present results of all statistical syntheses conducted. If meta-analysis was done, present for each the summary estimate and its precision (e.g. confidence/credible interval) and measures of statistical heterogeneity. If comparing groups, describe the direction of the effect.	Not applicable
	20c	Present results of all investigations of possible causes of heterogeneity among study results.	Results and Discussion
	20d	Present results of all sensitivity analyses conducted to assess the robustness of the synthesized results.	Not applicable
Reporting biases	21	Present assessments of risk of bias due to missing results (arising from reporting biases) for each synthesis assessed.	Results (Quality Assessment Across Studies)
Certainty of evidence	22	Present assessments of certainty (or confidence) in the body of evidence for each outcome assessed.	Discussion (narrative assessment)
DISCUSSION			
Discussion	23a	Provide a general interpretation of the results in the context of other evidence.	Discussion
	23b	Discuss any limitations of the evidence included in the review.	Discussion
	23c	Discuss any limitations of the review processes used.	Discussion
	23d	Discuss implications of the results for practice, policy, and future research.	Discussion
OTHER INFORMATION			
Registration and protocol	24a	Provide registration information for the review, including register name and registration number, or state that the review was not registered.	Not registered
	24b	Indicate where the review protocol can be accessed, or state that a protocol was not prepared.	Not applicable
	24c	Describe and explain any amendments to information provided at registration or in the protocol.	Not applicable
Support	25	Describe sources of financial or non-financial support for the review, and the role of the funders or sponsors in the review.	Acknowledgments
Competing interests	26	Declare any competing interests of review authors.	Conflict of Interest Statement
Availability of data, code and other materials	27	Report which of the following are publicly available and where they can be found: template data collection forms; data extracted from included studies; data used for all analyses; analytic code; any other materials used in the review.	Data not publicly available

Table 5: PRISMA 2020 Abstract Checklist

Section and Topic	Item	Checklist Item	Reported (Yes/No)
TITLE			
Title	1	Identify the report as a systematic review.	Yes
BACKGROUND			
Objectives	2	Provide an explicit statement of the main objective(s) or question(s) the review addresses.	Yes
METHODS			
Eligibility criteria	3	Specify the inclusion and exclusion criteria for the review.	Yes
Information sources	4	Specify the information sources (e.g. databases, registers) used to identify studies and the date when each was last searched.	Yes
Risk of bias	5	Specify the methods used to assess risk of bias in the included studies.	Yes
Synthesis of results	6	Specify the methods used to present and synthesise results.	Yes
RESULTS			
Included studies	7	Give the total number of included studies and participants and summarise relevant characteristics of studies.	Yes
Synthesis of results	8	Present results for main outcomes, preferably indicating the number of included studies and participants for each. If meta-analysis was done, report the summary estimate and confidence/credible interval. If comparing groups, indicate the direction of the effect (i.e. which group is favoured).	Yes
DISCUSSION			
Limitations of evidence	9	Provide a brief summary of the limitations of the evidence included in the review (e.g. study risk of bias, inconsistency and imprecision).	Yes
Interpretation	10	Provide a general interpretation of the results and important implications.	Yes
OTHER			
Funding	11	Specify the primary source of funding for the review.	Not applicable
Registration	12	Provide the register name and registration number.	Not applicable
